# Sirtuin-3 Protects Cochlear Hair Cells Against Noise-Induced Damage *via* the Superoxide Dismutase 2/Reactive Oxygen Species Signaling Pathway

**DOI:** 10.3389/fcell.2021.766512

**Published:** 2021-11-18

**Authors:** Wenqi Liang, Chunli Zhao, Zhongrui Chen, Zijing Yang, Ke Liu, Shusheng Gong

**Affiliations:** Department of Otolaryngology Head and Neck Surgery, Beijing Friendship Hospital, Capital Medical University, Beijing, China

**Keywords:** SIRT3, SOD2, noise-induced hearing loss, viral transduction, oxidative stress

## Abstract

Mitochondrial oxidative stress is involved in hair cell damage caused by noise-induced hearing loss (NIHL). Sirtuin-3 (SIRT3) plays an important role in hair cell survival by regulating mitochondrial function; however, the role of SIRT3 in NIHL is unknown. In this study, we used 3-TYP to inhibit SIRT3 and found that this inhibition aggravated oxidative damage in the hair cells of mice with NIHL. Moreover, 3-TYP reduced the enzymatic activity and deacetylation levels of superoxide dismutase 2 (SOD2). Subsequently, we administered adeno-associated virus-SIRT3 to the posterior semicircular canals and found that SIRT3 overexpression significantly attenuated hair cell injury and that this protective effect of SIRT3 could be blocked by 2-methoxyestradiol, a SOD2 inhibitor. These findings suggest that insufficient SIRT3/SOD2 signaling leads to mitochondrial oxidative damage resulting in hair cell injury in NIHL. Thus, ameliorating noise-induced mitochondrial redox imbalance by intervening in the SIRT3/SOD2 signaling pathway may be a new therapeutic target for hair cell injury.

## Introduction

Noise is a worldwide public health problem and an important risk factor for sensorineural hearing loss (SNHL). Oxidative stress-induced hair cell damage plays an important role in its development (Zhang et al., 2019). Noise exposure (NE) causes oxidative stress, elevates reactive oxygen species (ROS) levels, and causes hair cell damage, which further contributes to hearing loss (Delmaghani et al., 2015). Although several studies in the past decades have focused on countering noise-induced hair cell damage by interfering with ROS, there are no clinically relevant intervention targets to date. Therefore, key targets in the pathogenesis of noise-induced hearing loss (NIHL) should be explored.

Mitochondrial abnormalities caused by ROS accumulation play an important role in the pathogenesis of NIHL (Ding et al., 2020; Zhong et al., 2020). ROS are mainly produced in the mitochondrial oxidative respiratory chain; therefore, mitochondrial dysfunction can lead to cellular ROS accumulation (Gao et al., 2019; Zhao et al., 2019). Thus, mitochondrial dysfunction supposedly causes most oxidative damage (Tian et al., 2013). Sirtuin-3 (SIRT3), a member of the Sirtuin family, is a nicotinamide adenine dinucleotide (NAD^+^)-dependent deacetylase that is localized in the mitochondria (Onyango et al., 2002); it is the major mitochondrial deacetylase (Lombard et al., 2007) and a key factor regulating autophagy pathways. Autophagy and apoptosis are often simultaneously triggered by similar stimuli, such as oxidative stress, in both hair cells and spiral ganglion neurons (SGNs) (He et al., 2021; Liu et al., 2021). As the main site of cellular energy metabolism, mitochondria have various biological functions such as regulating cell proliferation, differentiation, apoptosis and senescence. SIRT3 regulates mitochondrial energy metabolism and biosynthesis; therefore, abnormal SIRT3 expression negatively affects mitochondrial function. Increased SIRT3 expression protects cells from oxidative stress-induced cell death and inhibits apoptosis in age-related SGNs and hair cells (Someya et al., 2010). Furthermore, SIRT3 overexpression reduces axonal degeneration induced by NE, thus making mice resistant to NIHL (Brown et al., 2014).

Superoxide dismutase 2 (SOD2) is a key antioxidant enzyme in mitochondria that reduces ROS production and protects cells from oxidative stress (Sarsour et al., 2014). Acetylation is one of the most important post-translational SOD2 modifications, leading to the downregulation of the SOD2 function (Dikalova et al., 2017). SIRT3 works by deacetylating proteins, particularly lysine of SOD2, to regulate its activity and thus maintain mitochondrial function (Yang et al., 2016).

Given the important role of mitochondrial function in the development of NIHL, we hypothesized that changes in SOD2 deacetylation levels due to SIRT3 activity are involved in the pathogenesis of NIHL. To test this hypothesis, we explored the protective effect of SIRT3 on hair cell injury in NIHL mice and its underlying mechanisms.

## Materials and Methods

### Animals and Treatments

**FIGURE 1:**
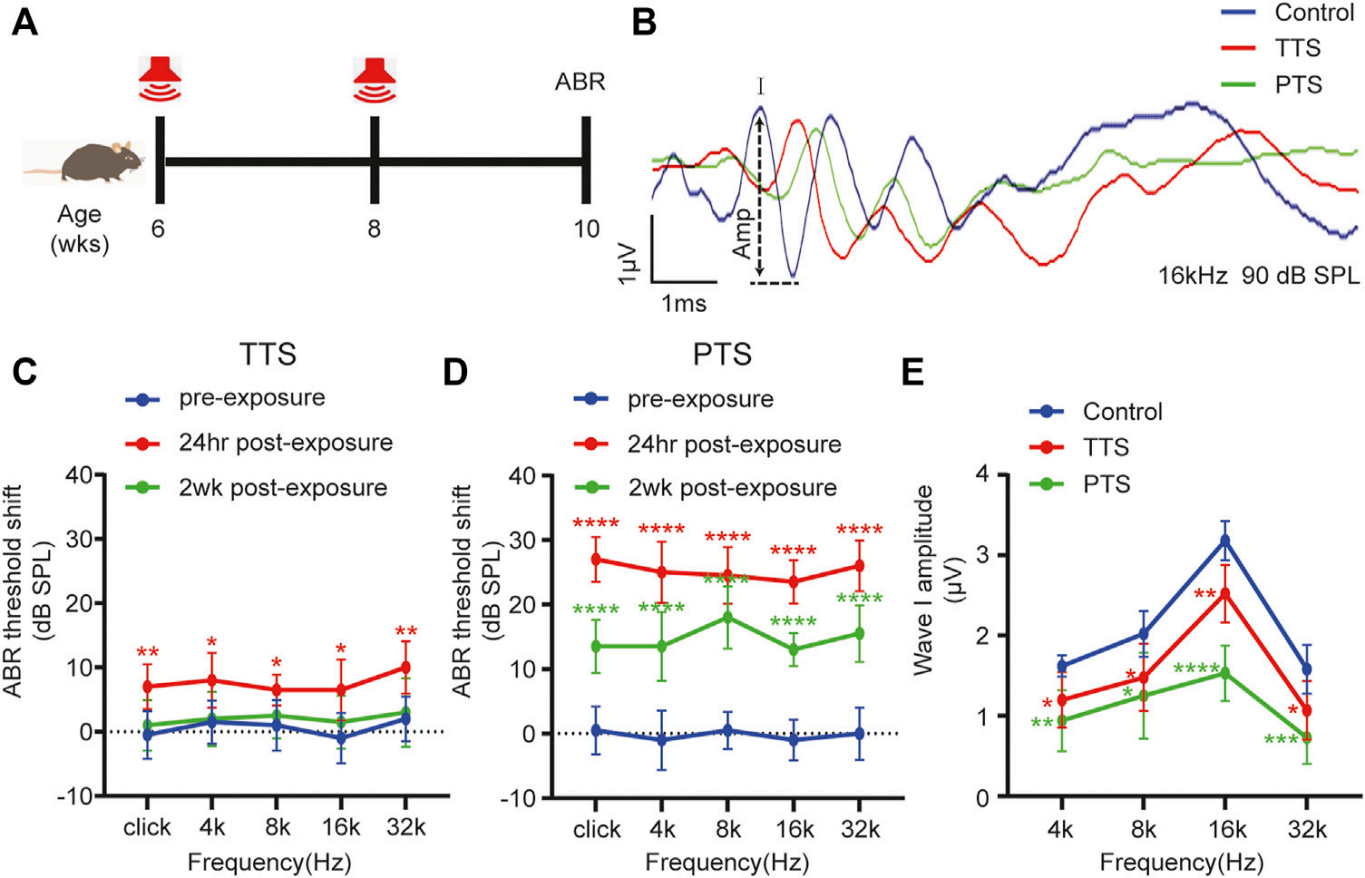
Changes in ABR thresholds and I-wave amplitudes after one or two NEs. **(A)** Mice underwent NE at 6 and 8 weeks of age or only at 8 weeks of age, and ABR testing was performed at 10 weeks of age. **(B)** Overlapped ABR waves were recorded in response to 16 kHz 90 dB SPL stimuli in control, TTS and PTS mice. **(C)** ABR test showing changes in the hearing thresholds of mice in the TTS group before NE and at 24 h and 2 weeks after NE. **(D)** ABR test showing changes in hearing thresholds of mice in the PTS group before NE and at 24 h and 2 weeks after NE. **(E)** ABR test showing changes in I-wave amplitude after NE. (*n* = 10 per group). ABR, auditory brainstem response; NE, noise exposure; TTS, temporary threshold shift; PTS, permanent threshold shift. *p < 0.05; **p < 0.01; ***p < 0.001; ****p < 0.0001.

C57BL/6 J male mice were obtained from the Experimental Animal Center of Capital Medical University (Beijing, China). Experiments were performed on mice after abnormal hearing was excluded by audiometric testing. According to the different interventions strategies, mice were randomly divided into the following groups: 27 mice in the control group, 5 in the TTS group, 73 in the PTS group, 37 in the PTS+3-TYP group, 5 in the PTS + corn oil group, 5 in the PTS+2-ME group, 5 in the PTS + saline group, 9 in the PTS + AAV-SIRT3 group, 5 in the PTS + AAV-GFP group, and 5 in the PTS+2-ME + AAV-SIRT3 group. Mice in the PTS group received NE at 6 and 8 weeks old of mice, whereas mice in the TTS group received NE only at 8 weeks old, and both groups underwent auditory brainstem response (ABR) testing at 10 weeks (Figure 1A). For the intervention group mice received 3-TYP, 2-ME or AAV-SIRT3 in addition to NE. Both 3-TYP (MCE Chemicals & Equipment Co., Malta, NY) and 2-methoxyestradiol (2-ME; Selleck Chemicals, Houston, TX) were administered by intraperitoneal injection starting 1 week prior to NE for 7 days 3-TYP was administered at 50 mg/kg/day, and 2-ME was administered at 16 mg/kg/day. The control group mice were injected with equal amounts of either saline or corn oil. AAV-SIRT3 was surgically introduced into the inner ear at 4 weeks in the AAV-SIRT3 intervention group of mice, and an equal amount of empty virus was introduced into the control group mice. The animals were cared for and used in accordance with the Guideline for the Care and Use of Laboratory Animals of the National Institutes of Health. The experimental protocols were approved by the Committee on the Ethics of Animal Experiments of Capital Medical University.

### Noise Exposure

Animals in the noise group were placed in wire mesh cages inside an anechoic chamber and exposed to 100 dB sound pressure level (SPL) broadband white noise for 2 h. Noise synthesis was performed using Cool Edit Pro software (Adobe Systems, San Jose, CA) and transmitted through XTi4002, CROWN amplifiers (Harman, Elkhart, IN) to two speakers (JBL KP6000, PROFESSIONAL, Harman) for noise release.

### ABR Testing

We used the TDT system 3 evoked potential workstation (Tucker-Davis Technologies, Alachua, FL, United States) to record ABR. Prior to initiating the ABR test, mice were anesthetized by intraperitoneal injection of 100 mg/kg ketamine and 10 mg/kg xylazine. After anesthesia, the recording electrode was inserted in the subcutaneous area at the midpoint of the line connecting the anterior margins of the auricles on both sides of the mouse, the reference electrode was inserted into the subcutaneous area behind the tested ear, and the ground electrode was inserted into the subcutaneous area behind the contralateral ear. The tests were performed using click and tone bursts at frequencies of 4, 8, 16, and 32 kHz with the SigGenRZ software (Tucker-Davis Technologies). Sound intensity was attenuated from 90 to 0 dB in 5 dB intervals, and the responses were analyzed using BioSigRZ software (Tucker-Davis Technologies), digitized, and averaged for each frequency-level combination (1,024 samples/level). The threshold was defined as the lowest stimulus decibel that evoked a significant positive wave in the response trajectory. All ABR tests were performed by the same researchers.

### Tissue Preparation

After ABR testing, mice were sacrificed under deep anesthesia, and the cochlea was removed and immersed in 4% paraformaldehyde at 4°C for overnight fixation. Parts of the cochlea were decalcified in 10% ethylenediaminetetraacetic acid (EDTA) for 12 h and subsequently dehydrated in 30% sucrose for 2 h. Afterward, the cochlea was immersed in an optimal cutting temperature compound. Frozen sections of 10 µm thickness were stored at −20°C for immunohistochemistry. Other cochleae were decalcified using 10% EDTA for 2 h; structures such as vascular striae, spiral ligaments and capping membranes were carefully excised under a microscope, and the remaining basilar membranes were divided into the apical, middle, and basal turns for immunofluorescence staining.

### Immunostaining

Cochlear sections and spreads were incubated with 5% normal goat serum (ZSGB-BIO, Beijing, China) and 0.3% Triton X-100 (Sigma-Aldrich, St. Louis, MO) in phosphate-buffered saline (PBS) for 2 h at room temperature. After washing three times with PBS, the samples were incubated with primary antibody solution at 4°C overnight. After multiple washes, the samples were incubated with secondary antibodies at a ratio of 1:300 for 2 h at room temperature and protected from light. The primary antibodies used were anti-myosin-VIIa (1:300, *Proteus* BioSciences Inc. Ramona, CA), anti-CtBP2 (1:500, BD Biosciences, Franklin Lakes, NJ), anti-GluR2 (1:400, Millipore, Burlington, MA), anti-8-hydroxy-2′-deoxyguanosine (8-OHdG, 1:300, Abcam, Cambridge, United Kingdom), anti-green fluorescent protein (GFP) (1:100, Santa Cruz Biotechnology, Dallas, TX), and anti-4-HNE (1:500; Abcam). The secondary antibodies used were goat anti-mouse IgG1 Alexa Fluor 568, goat anti-mouse IgG2a Alexa Fluor 488, and goat anti-rabbit IgG (H + L) Alexa Fluor 647 (1:300, Invitrogen/Molecular Probes, Eugene, OR). 4, 6-diamidino-2-phe-nylindole (DAPI) was used for the final addition of coverslips. The expression of 8-OHdG and 4HNE was analyzed using Image-Pro Plus 6.0 software (Media Cybernetics, Inc. United States).

### Hair Cells and Synapses Counting

Cochlear samples were observed and imaged using a Leica scanning confocal microscope (Leica Camera AG, Hessen, Germany). The basilar membrane was divided into apical, middle, and basal turns to count the hair cells separately. Lost hair cells were examined under a ×63 oil immersion objective lens and their numbers and proportions were statistically analyzed. Ten cochlear samples from each group were used for hair cell counts. Scans were taken at 0.35 μm/layer intervals from the top to the bottom of the inner hair cell (IHC) and subsequently superimposed. In each region, the total number of synapses was evaluated for a total of approximately 10 IHCs, and the average was subsequently calculated. The number of paired and unpaired synapses at the apical, middle, and basal turns were counted.

### Viral Constructs and Posterior Semicircular Canal Transduction

Purified adeno-associated virus (AAV) eight vectors with SIRT3 and the GFP gene (AAV8-SIRT3-GFP) and AAV8-GFP vectors were obtained from Vigenebio Biosciences Co. (Jinan, China). The expression of carrier genes was driven by the cytomegalovirus promoter. Viral particles were purified using ion-exchange column chromatography; physical titers were 1.81 × 10^12^ vg/ml (AAV8-SIRT3-GFP) and 1.19 × 10^12^vg/mL (AAV8-GFP). The vectors were stored at approximately −80°C. As previously described, the injection was administered through the semicircular canals (canalostomy) (Guo et al., 2018). A 2 µl volume of the virus was injected at a rate of 0.5 μl/min.

### Western Blotting (WB)

Cochlear proteins were extracted and protein concentrations were measured using a BCA Protein Quantification Kit (Beyotime, China). Equal amounts of proteins were separated by 12% sodium dodecyl sulfate polyacrylamide gel electrophoresis and subsequently electrotransferred to polyvinylidene fluoride membranes. After blocking in 5% skim milk at room temperature for 1 h, samples were incubated with anti-SOD2(Cell Signaling Technology), anti-ac-SOD2 (Abcam), anti-C-cas3 (Cell Signaling Technology), anti-Cyt c (Abcam), anti-COX IV (Abcam), and β-actin (Cell Signaling Technology) primary antibodies overnight at 4°C, followed by incubation with the appropriate secondary antibody at room temperature for 1 h. After washing with Tris-buffered saline with Tween, protein bands were observed using chemiluminescent reagents (Applygen Technologies Inc. China).

### ROS Detection

The intracellular ROS assay was performed using the Reactive Oxygen Species Assay Kit (Beyotime, Shanghai, China). After collecting the cochlear cells, they were resuspended in 100 µL of diluted dichloro-dihydro-fluorescein diacetate and incubated for 20 min at 37°C in a cell culture incubator. Afterward, the cells were washed three times with a serum-free cell culture medium, and chemiluminescence was measured using an EnSpire enzyme marker (PerkinElmer, Waltham, MA).

A mitochondrial membrane potential assay kit with JC-1 (Beyotime, China) was used to analyze mitochondrial ROS production. The cochlea was cut and digested with trypsin, and the cells were collected by centrifugation. Cell pellets were then resuspended in a cell culture medium containing JC-1 staining working solution and incubated at 37°C for 20 min in the dark, washed with cold JC-1 staining buffer and analyzed by flow cytometry (FACS Aria IIu, BD Biosciences) within 1 h.

### Measurement of SOD2 Activity

The SOD2 activity assay was performed using the Cu/Zn-SOD and Mn-SOD Assay Kit with WST-8 (Beyotime, China) according to the manufacturer’s instructions. After the cochlear tissue was cut and digested with trypsin, the cells were extracted by centrifugation. Cells were incubated for 1 h at 37°C using Cu/Zn-SOD inhibitor A and for 15 min at 37°C using Cu/Zn-SOD inhibitor B. Consequently, samples were added to a 96-well plate and mixed with the assay solution. Chemiluminescence was measured using an EnSpire enzyme marker (PerkinElmer). SOD2 viability units were calculated using a standard curve.

### Determination of NADPH Oxidase Activity

According to the manufacturer’s instructions, NADPH oxidase activity was assayed using a NADP +/NADPH Assay Kit with WST-8 (Beyotime, China). Bilateral cochleae were dissected from eight mice (four per group) and homogenized in NADP +/NADPH extracts. The samples were centrifuged at 12,000 g for 10 min at 4 °C, and the supernatant was subsequently collected. For testing, 200 μl of the sample was aspirated and placed in a water bath at 60°C for 30 min. Afterward, they were centrifuged at 10,000 g for 5 min at 4°C, the supernatant was collected and mixed with G6PDH working solution and color development solution, and incubated for 20 min at 37°C protected from the light. Sample chemiluminescence was measured using an EnSpire enzyme marker (PerkinElmer). NADPH oxidase activity was calculated from the standard curve.

### Statistical Analysis

Quantitative values are expressed as mean ± standard error of the mean (SEM) and statistically analyzed using GraphPad Prism software version 8.0 (GraphPad Software Inc., San Diego, CA). The statistical methods selected were two-way analysis of variance or unpaired *t*-test, as appropriate. For all analyses, values of *p* < 0.05 were considered statistically significant.

## Results

### Noise Exposure Causes a Hearing Threshold Shift in Mice

Using the ABR test, we found that the hearing thresholds of mice receiving a single NE increased transiently after NE and then decreased, which were not significantly different from those of the control group mice at 2 weeks after NE, indicating that a single NE caused a hearing temporary threshold shift (TTS) (Figure 1C). The hearing thresholds in mice increased after two NEs, and although they decreased 2 weeks later, there was still a significant difference from the thresholds of the control group mice, indicating that two NEs caused a permanent threshold shift (PTS) in hearing in mice (Figure 1D). The amplitude of ABR wave I in both the PTS and TTS groups was significantly different from that in the control group (Figures 1B,E).

### Noise Exposure Leads to Loss of Cochlear Outer Hair Cells and Reduced Inner Hair Cell Synapses

**FIGURE 2:**
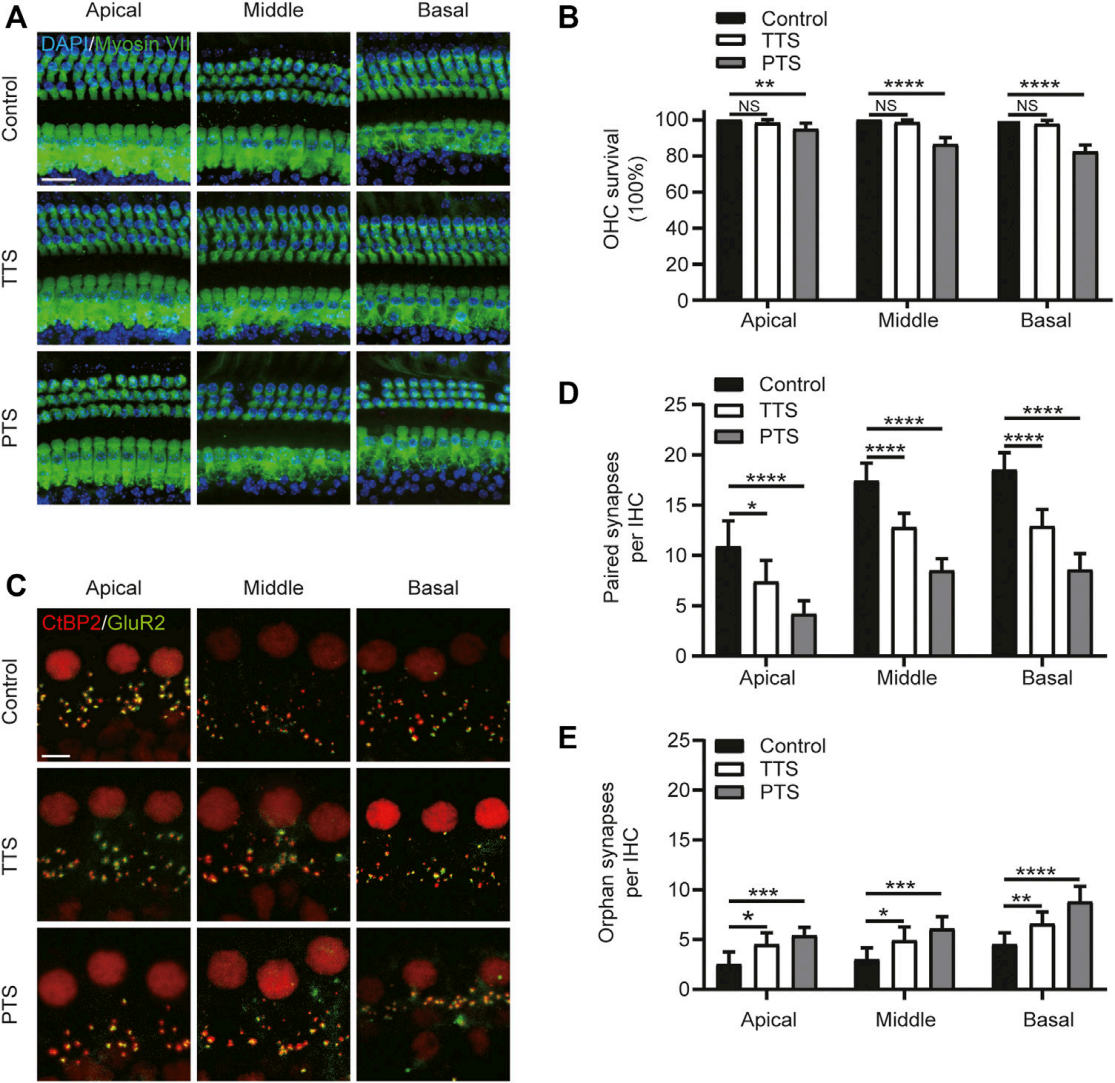
Morphological alterations of mice in the PTS and TTS groups. The basilar membrane was divided into apical, middle, and basal turns for separate observation. **(A)** Immunohistochemical staining showing changes in the number of hair cells (Myosin VII, green; DAPI, blue) in the PTS and TTS groups. **(B)** Quantification of Myosin VII and DAPI-positive HCs. **(C)** Immunohistochemical staining showing changes in the number of presynaptic (CtBP2, red) and postsynaptic structures (GluR2, green) in the IHCs of the PTS and TTS groups. **(D)** Quantification of CtBP2 and GluR2 overlapping fluorescent spots. **(E)** Quantification of CtBP2 or GluR2 fluorescent spots alone. (*n* = 10 per group). TTS, temporary threshold shift; PTS, permanent threshold shift; DAPI, 4, 6-diamidino-2-phenylindole; OHC, outer hair cell, IHC, inner hair cell. NS, not significant; *p < 0.05; **p < 0.01; ***p < 0.001; ****p < 0.0001. Scale bars: A, 20 μm; C, 5 μm.

We examined changes in the number of hair cells and synapses after NE. We divided the cochlear Basilar membrane into the apical, middle, and basal turns in the PTS and TTS group mice for this study. Compared with the control group mice, the TTS group mice showed no significant loss of outer hair cells (OHCs) in either the apical, middle or basal turns, whereas the PTS group mice showed significant OHC loss (Figures 2A,B). We analyzed the synapses of the IHCs in two dimensions: paired and orphan synapses. Compared with the control group mice, both the PTS and TTS group mice had decreased number of paired synapses, with the PTS group mice showing a more significant decrease. Both the PTS and TTS groups showed an increased number of unpaired synapses compared to the control group, with a greater increase observed in the PTS group (Figures 2C–E). Regarding both audiology and morphology, the damage in the PTS group was more significant than that in the TTS group; therefore, we chose the PTS group to investigate the mechanism of SIRT3 in NIHL.

### Noise Exposure Induces Oxidative Damage in Hair Cells

**FIGURE 3:**
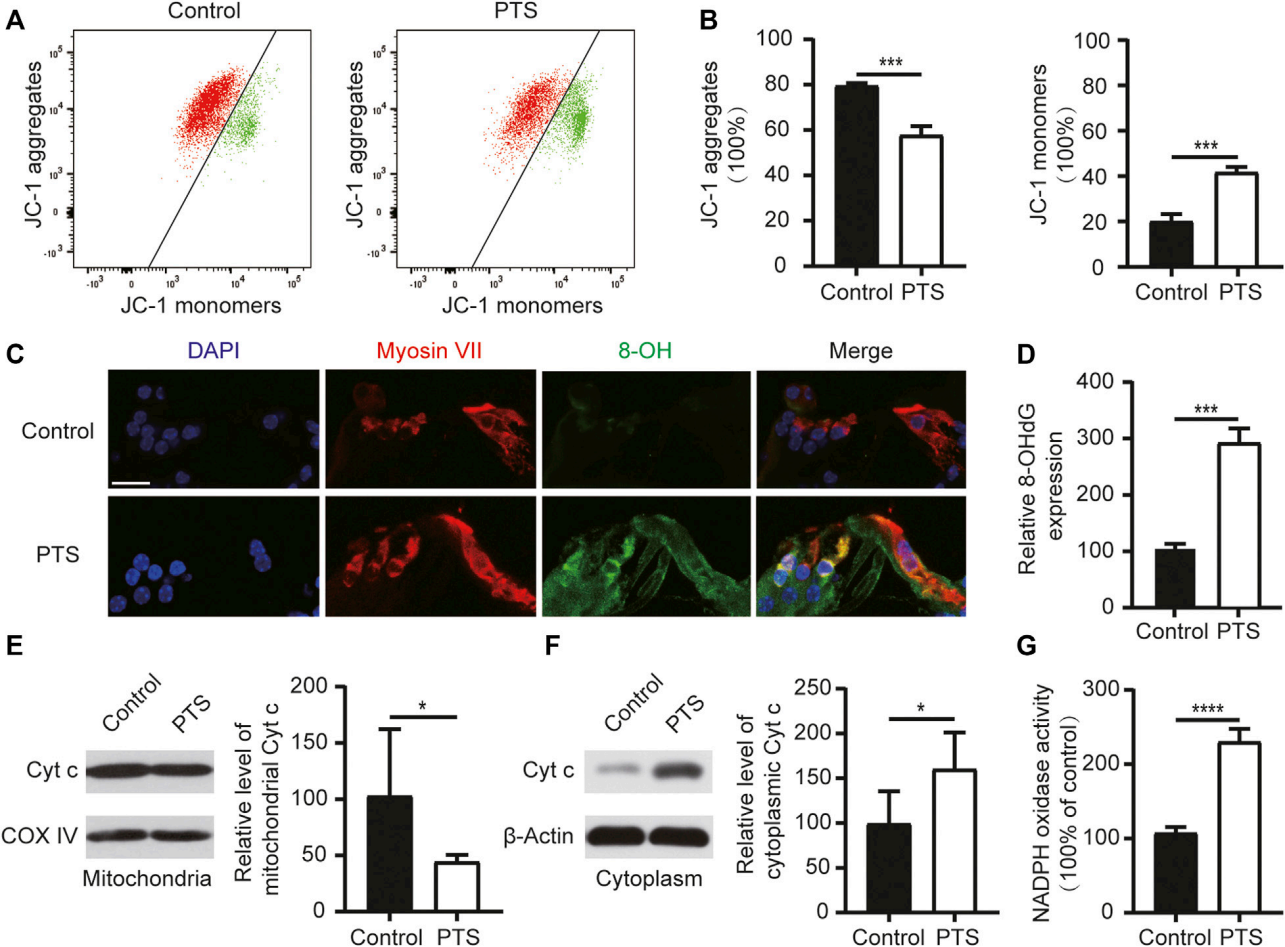
Changes in mitochondrial membrane potential, ROS, apoptosis levels, and NADPH oxidase activity in the hair cells of mice in the PTS and control groups. **(A)** Detection of mitochondrial membrane potential in the PTS and control groups using flow cytometry. **(B)** Ratio of JC-1 aggregates and JC-1 monomers (*n* = 3 per group). **(C)** Immunohistochemical staining of frozen sections of the cochlea showing changes in 8-OH expression in the PTS and control groups. **(D)** Quantification of 8-OH fluorescence intensity (*n* = 5 per group). **(E)** WB showing changes in Cytochrome c expression in the mitochondria. **(F)** WB showing changes in Cytochrome c expression in the cell plasma (*n* = 6 per group). **(G)** Levels of NADPH oxidase activity in the different groups (*n* = 4 per group). ROS, reactive oxygen species; PTS, permanent threshold shift; WB, western blotting. *p < 0.05; ***p < 0.001; ****p < 0.0001. Scale bars: C, 20 μm.

We analyzed the mitochondrial membrane potential in the inner ear cells of the PTS and control group mice. Compared with the control group mice, the PTS group mice showed decreased JC-1 aggregates and increased JC-1 monomers, indicating that the mitochondrial membrane potential was decreased in these mice (Figures 3A,B). Immunofluorescence staining of frozen sections of mouse cochlea revealed that 8-OH expression was elevated in the PTS group compared to that in the control group (Figures 3C,D). WB analysis on mouse cochleae revealed that Cytochrome c expression was decreased in the mitochondria and increased in the cytoplasm in the PTS group compared with that in the control group (Figures 3E,F). The level of NADPH oxidase activity in the PTS group was significantly higher than that in the control group (Figure 3G).

### 3-TYP Exacerbates Noise-Induced Hair Cell Damage

**FIGURE 4:**
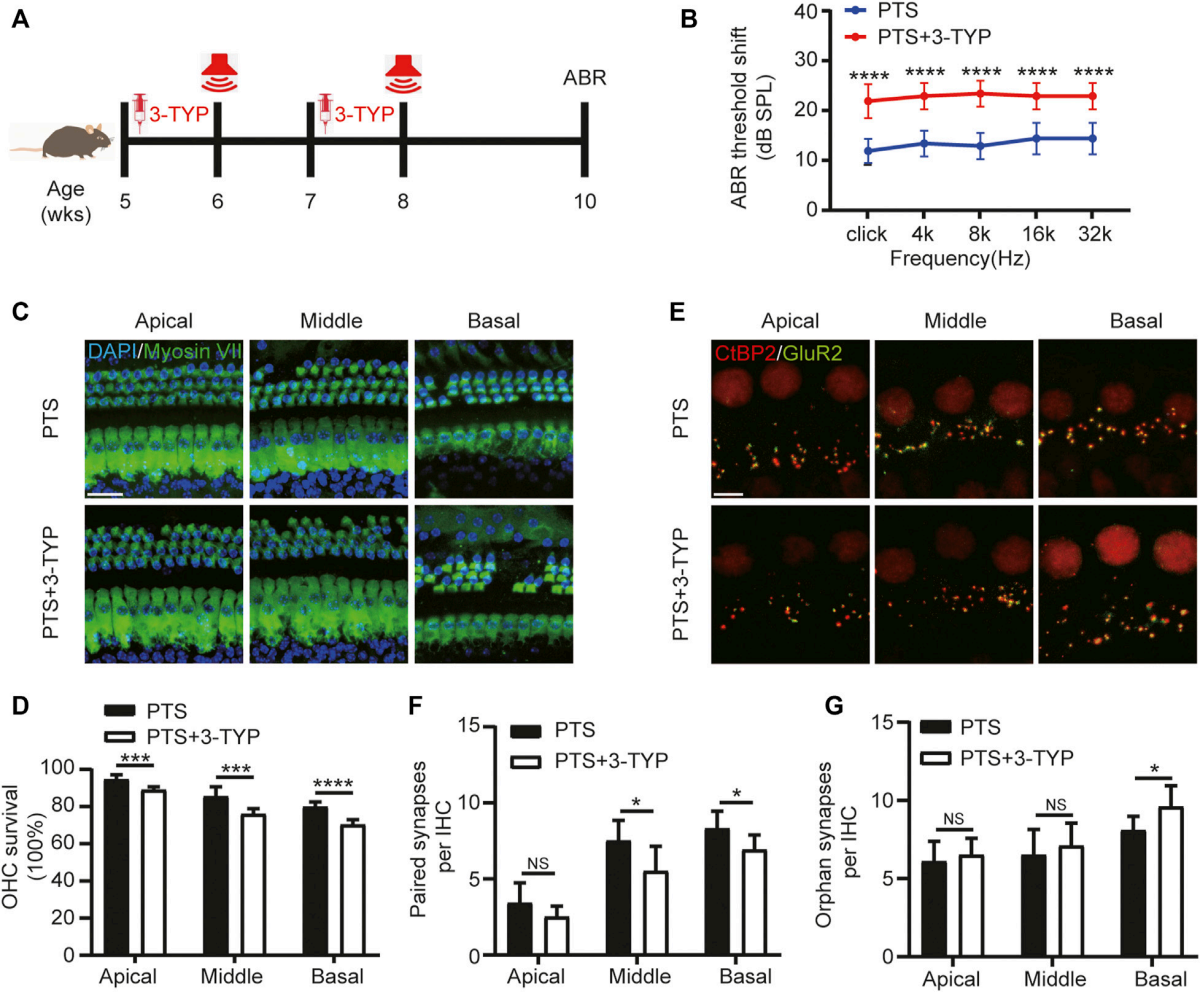
Effect of SIRT3 inhibitors on hair cells in the PTS group. **(A)** Schedule of 3-TYP and NE administration to C57BL/6 J mice. **(B)** ABR findings showing the effect of 3-TYP on the hearing threshold in mice after NE. **(C)** Immunohistochemical staining showing 3-TYP-induced changes in the number of hair cells (Myosin VII, green; DAPI, blue) after NE. **(D)** Quantification of Myosin VII and DAPI-positive HCs. **(E)** Immunohistochemical staining showing the effect of 3-TYP on the number of presynaptic (CtBP2, red) and postsynaptic (GluR2, green) structures after NE. **(F)** Quantification of CtBP2 and GluR2 overlapping fluorescent spots. **(G)** Quantification of CtBP2 or GluR2 fluorescent spots alone. (*n* = 10 per group). PTS, permanent threshold shift; NE, noise exposure; ABR, auditory brainstem response; DAPI, 4, 6-diamidino-2-phenylindole; OHC, outer hair cell, IHC, inner hair cell. NS, not significant; *p < 0.05; ***p < 0.001; ****p < 0.0001. Scale bars: C, 20 μm; E, 5 μm.

We used the SIRT3 inhibitor 3-TYP for the intervention, which was administered 1 week before each NE (Figure 4A). The ABR test results showed that the hearing threshold of the PTS+3-TYP group was significantly higher than that of the PTS group (Figure 4B). The OHC survival rate was significantly lower in the PTS+3-TYP group than in the PTS group (Figures 4C,D). The analysis of the number of paired synapses at the level of IHCs revealed no significant changes in the basolateral membrane in the PTS+3-TYP group compared with that in the PTS group, while this number was significantly reduced in the middle and basal turns. The number of unpaired synapses in the PTS+3-TYP and PTS groups was not significantly different in the apical and middle turns, whereas a significantly higher value was observed in the basal rotation (Figures 4E–G).

### 3-TYP Increases the Acetylation Level of SOD2 and Aggravates Oxidative Stress and Apoptosis

**FIGURE 5:**
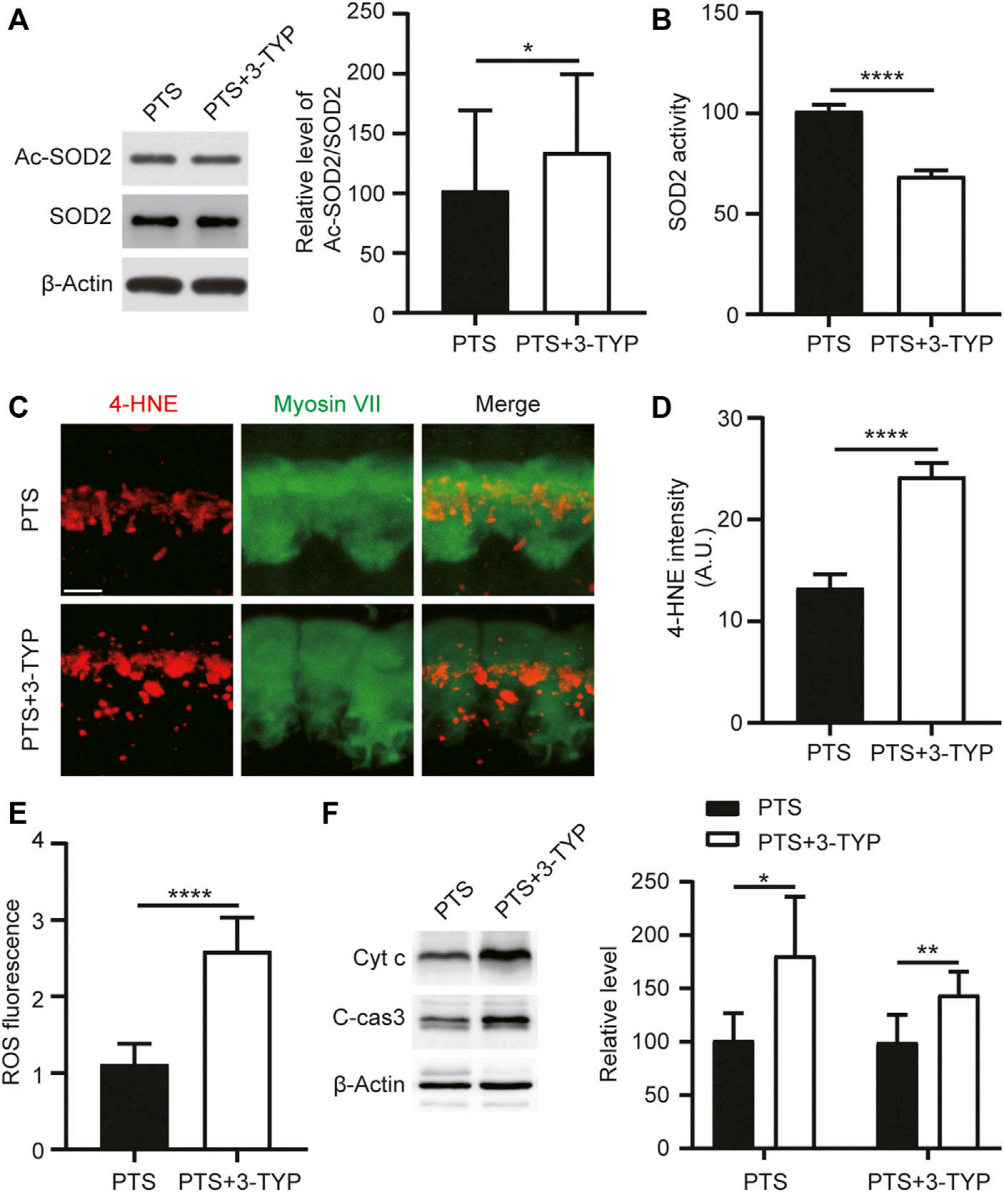
Effect of 3-TYP on SOD2 acetylation, ROS and apoptosis-related protein levels in the hair cells of mice in the PTS group. **(A)** Western blotting showing the effect of 3-TYP on SOD2 acetylation levels in mice in the PTS group. The results are expressed as the percentage of the PTS group, which was set to 100% (*n* = 3 per group). **(B)** Effect of 3-TYP on SOD2 activity in the cochlear cells of PTS mice (*n* = 10 per group). **(C)** Immunohistochemical staining showing changes in 4-HNE fluorescence intensity in IHCs. **(D)** Quantification of 4-HNE fluorescence intensity (*n* = 6 per group). **(E)** Detection of ROS levels in the cochlear cells (*n* = 10 per group). **(F)** WB showing changes in caspase 3 and Cytochrome c expression (*n* = 6 per group). ROS, reactive oxygen species; PTS, permanent threshold shift; IHC, inner hair cell. *p < 0.05; ****p < 0.0001. Scale bars: C, 5 μm.

To explore the effect of 3-TYP on SOD2, we performed WB assays for both Ac-SOD2 and SOD2 in the cochlea and assayed SOD2 activity. We observed that the acetylation level of SOD2 was elevated in the PTS+3-TYP group compared to that in the PTS group (Figure 5A). SOD2 activity was significantly decreased in the PTS+3-TYP group compared with that in the PTS group (Figure 5B). 4-Hydroxynonenal (4HNE) was used to detect cellular oxidative stress levels, and we observed that 4HNE expression in IHCs was significantly higher in the PTS+3-TYP group than in the PTS group (Figures 5C,D). In the cochlea, intracellular ROS levels were significantly higher in the PTS+3-TYP group than in the PTS group (Figure 5E). Caspase 3 and Cytochrome c protein expression levels were significantly higher in the PTS+3-TYP group than in the PTS group (Figures 5F,G).

### SIRT3 Overexpression Protects Hair Cells Against Noise Exposure in a SOD2 Dependent Manner

**FIGURE 6:**
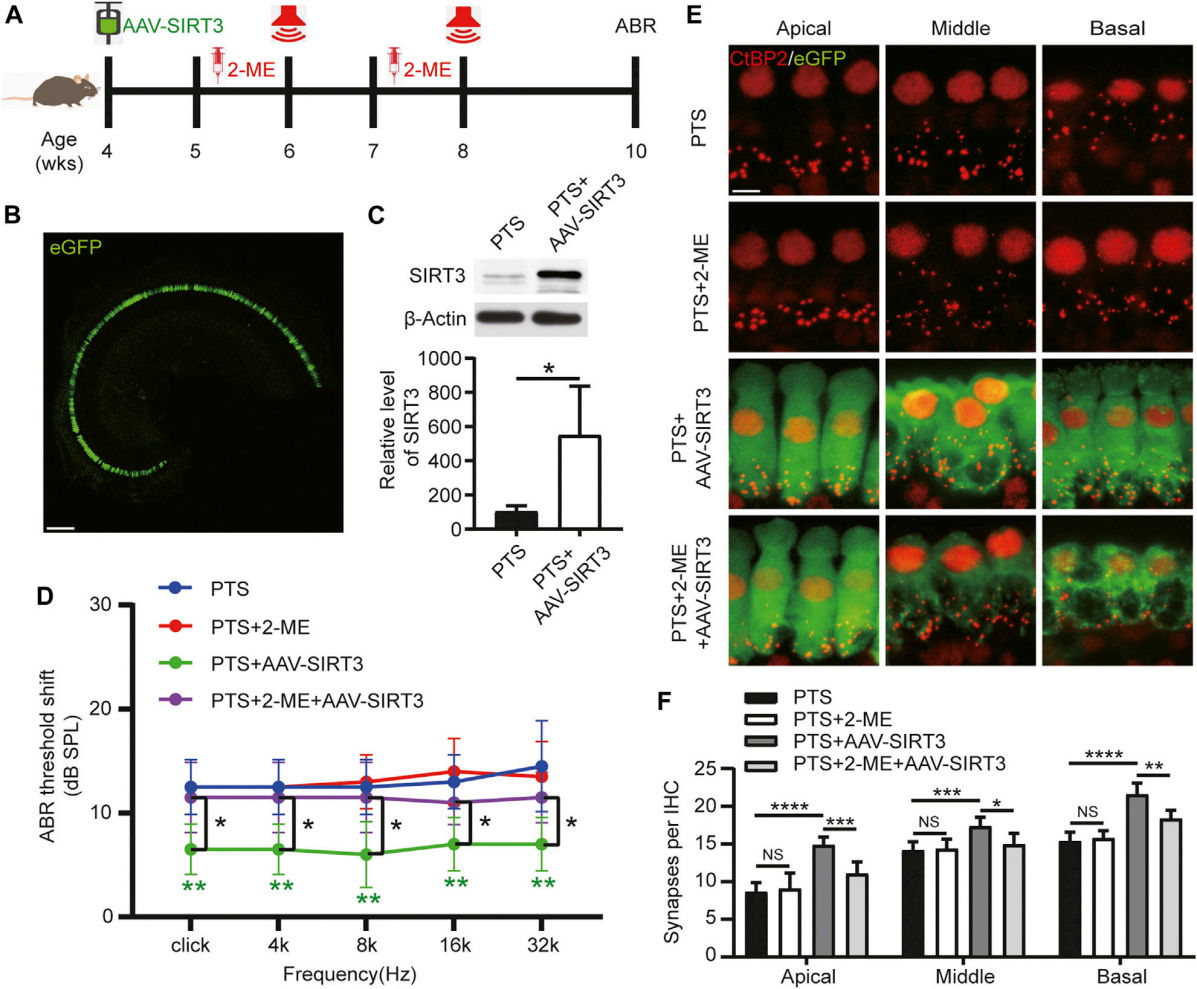
Effect of AAV-SIRT3 and SOD2 inhibitors on the hair cells of mice in the PTS group. **(A)** Schedule of AAV-SIRT3 and SOD2 inhibitor 2-ME administration to C57BL/6 J mice. **(B)** Representative confocal images of the cochlear apical turn via the posterior semicircular canal after injecting 2 μL of AAV-SIRT3. **(C)** WB showing changes in SIRT3 expression after AAV-SIRT3 injection. (n = 4 per group). **(D)** ABR showing the effects of AAV-SIRT3 and 2-ME on hearing thresholds in PTS mice. **(E)** Immunohistochemical staining showing the effects of AAV-SIRT3 and 2-ME on the number of presynaptic structures (CtBP2, red) in mice after NE. **(F)** Quantification of CtBP2 fluorescent spots. (*n* = 10 per group). AAV, adeno-associated virus; PTS, permanent threshold shift; 2-ME, 2-methoxyestradiol; ABR, auditory brainstem response; NE, noise exposure. NS, not significant; *p < 0.05; **p < 0.01; ***p < 0.001; ****p < 0.0001. Scale bars: B, 100 μm; E, 5 μm.

We induced SIRT3 overexpression by introducing AAV-SIRT3 in 4-week-old mice undergoing posterior semicircular canal surgery. The SOD2 inhibitor 2-ME was administered starting 1 week before each NE (Figure 6A). Most IHCs could be transfected with AAV-SIRT3, as observed on confocal microscopy (Figure 6B). WB results showed that SIRT3 expression was significantly higher in the PTS + AAV-SIRT3 group than in the PTS group (Figure 6C). These findings indicate that SIRT3 overexpression by the introduction of AAV-SIRT3 into the posterior semicircular canal is feasible. The ABR results showed no significant difference in hearing thresholds between the PTS and PTS+2-ME groups, and hearing thresholds were significantly lower in the PTS + AAV-SIRT3 group. The thresholds were significantly higher in the PTS+2-ME + AAV-SIRT3 group than in the PTS + AAV-SIRT3 group (Figure 6D). Immunofluorescence CtBP2 staining in the apical, middle, and basal turns revealed no significant difference in the number of synapses in the PTS+2-ME group compared with that in the PTS group, whereas the number of synapses in the PTS + AAV-SIRT3 group was significantly elevated. Compared with the PTS + AAV-SIRT3 group, the number of synapses in the PTS+2-ME + AAV-SIRT3 group was significantly decreased in the apical, middle, and basal turns (Figures 6E,F). The audiological and morphological levels were not significantly different between the PTS + AAV8-GFP and PTS group mice; therefore, these findings are not shown in this figure.

## Discussion

NIHL is a major occupational risk in industrialized countries and is estimated to affect approximately 5% of the world population (Basner et al., 2014). In the inner ear, the mechanical vibration of a sound wave is transduced into electrical signals by hair cells (Chen et al., 2021), and these electrical signals are transmitted to auditory cortex through the synapses of the SGNs (Guo et al., 2020). Loss of hair cells and SGNs is the main cause of hearing loss (Zhang et al., 2021). It is well documented that oxidative damage is a major cause of hearing loss cochlear and hair cells, which can be easily damaged by various insults, including mutations in deafness genes (Qian et al., 2020), aging (Qi et al., 2019), noise, drugs, infections and injuries (Liu et al., 2019), or lack of regenerative capacity (Chen et al., 2021). NE increases ROS level, which causes hearing loss through oxidative damage to hair cells and neurons (Delmaghani et al., 2015). Mitochondria are the main sites of intracellular ROS production. We found that mitochondrial SIRT3 plays an important role in NIHL by regulating redox imbalance through SOD2 activation.

In C57BL/6 J mice, repetitive noise-induced impairment of cochlear function and altered synaptic morphology have dose-dependent characteristics (Qian et al., 2021). Our previous study found that a single moderate NE caused TTS; however, repeated NE caused PTS (Luo et al., 2020). To select a suitable model for NIHL, we chose 2 h of 100 dB SPL white noise in one and two episodes of NE. One NE caused TTS, and two NEs caused PTS (Figure 1). Previous studies have shown that the main site of noise-induced inner ear damage is the ribbon synapse (Feng et al., 2020). Therefore, in addition to OHC count, we counted IHC ribbon synapses. In agreement with previous studies, we found that hair cell damage was more significant in the PTS group than in the TTS group, both at the audiological and morphological levels; therefore, we chose the PTS group for further study (Figure 2). Oxidative stress plays an important role in NIHL, and genetic variants of oxidative stress affect the susceptibility to noise (White, 2019). We found that, compared with the control group mice, the PTS group mice had significantly reduced mitochondrial membrane potential and significantly increased 8-OH expression, indicating that NE caused intracellular ROS accumulation by affecting mitochondrial function (Figure 3).

Studies have reported that SIRT3 can balance ROS levels by modifying post-translational levels and can activate long-term transcriptional programs to protect cells from oxidative damage (van de Ven et al., 2017). SIRT3 resists the ototoxicity of gentamicin (Han et al., 2020), and its upregulation protects mice against hearing loss caused by high-fat diet and aging (Miwa, 2021). SIRT3 is a major regulator of the mitochondrial oxidative stress response (Giblin et al., 2014). Nicotinamide riboside (NR), as a SIRT3 agonist, has a protective effect on hair cells and synapses in mice after NE by reducing cellular oxidative damage (Han et al., 2020). Further, supplementation with the NAD^+^ precursor NR to elevate NAD levels can rescue animals from NIHL; however, animals lacking SIRT3 do not benefit from this effect (Brown et al., 2014). This is consistent with our findings. Using 3-TYP to suppress SIRT3, we found that SIRT3 inhibition significantly aggravated the damage to hair cells and worsened hearing loss in mice after NE (Figure 4). Consistent with previous studies, we suggest that SIRT3 plays a protective role against NE-induced hair cell damage.

SOD2, a SOD that is expressed only in the intracellular mitochondrial matrix (Slot et al., 1986), plays a crucial role in resisting oxidative damage caused by mitochondrial superoxide (Jowko et al., 2017). SOD2 serves as the first line of defense against mitochondrial oxidative damage and is the main mitochondrial ROS scavenger (Miao and St, 2009). SOD2 converts SOD to hydrogen peroxide, which is subsequently converted to water by catalase and other peroxidases (Fridovich, 1995). Diet-induced obese mice showed significant hearing loss due to reduced SOD2 levels, resulting in elevated ROS and increased hair cell mortality (Lee et al., 2020). SOD2 upregulation plays a protective role in acute acoustic injury in rats (Zhu et al., 2020). SOD2 is post-translationally regulated in several ways; however, acetylation is the major SOD2 active modification (Zou et al., 2016). The reduced level of lysine acetylation in SOD2 increases its enzymatic activity (Candas and Li, 2014). The 4-HNE level reflects cellular membrane damage due to lipid peroxidation induced by ROS. We found that SIRT3 inhibition resulted in a significant increase in the acetylation level of SOD2, a decrease in SOD2 activity, a significant increase in ROS levels, and an increase in apoptosis (Figure 5).

SIRT3 maintains ROS homeostasis by activating SOD2 through deacetylation and converting harmful superoxide radicals to harmless oxygen or hydrogen peroxide (Shen et al., 2020). In multiple disease models, SIRT3 has been reported to play an important role in antioxidant damage by regulating its downstream molecule, SOD2. In a mouse model of acute kidney injury, SIRT3 reduced Ac-SOD2 and ROS levels and attenuated oxidative damage, thereby resisting apoptosis (Zhang et al., 2021). Activating the Akt-SIRT3-SOD2 signaling pathway ameliorates mitochondrial damage and attenuates brain ischemia-reperfusion injury in diabetic mice (Liu et al., 2021). In this study, we found that SIRT3 overexpression attenuated hearing loss by protecting hair cells, and this protective effect could be blocked by the SOD2 inhibitor 2-ME (Figure 6). Therefore, we conclude that SIRT3 overexpression reduces noise-induced hair cell damage by activating SOD2. Mitochondria, as a major source of ROS, play a key role in regulating cellular functions. It is widely believed that mitochondrial oxidative stress leads to NIHL. Our current work reveals innovative molecular mechanisms by which mitochondrial oxidative stress regulates noise-induced hair cell injury. Thus, the study results provide new potential therapeutic targets with important implications for intervention in the development of NIHL.

However, our study has some limitations. First, mitochondrial redox homeostasis is a complex system, and the linkage of SIRT3/SOD2 signaling with other antioxidant systems requires further investigation. Second, further investigations are warranted to evaluate SIRT3/SOD2-mediated alterations in the mitochondrial function of hair cells and determine the pathogenesis of TTS.

## Conclusion

**FIGURE 7:**
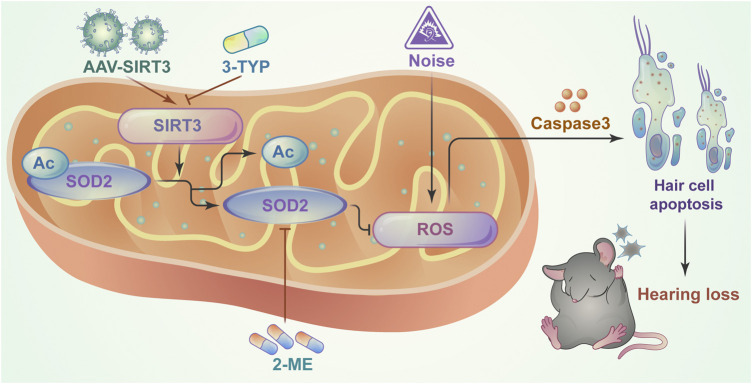
Schematic model showing the critical role of mitochondrial oxidative stress in NIHL model hair cells and the protective role of SIRT3/SOD2 signaling. SIRT3 deacetylates and activates SOD2, reducing reactive oxygen species production and thereby ameliorating noise-induced hair cell damage.

This study identified a critical role for the SIRT3/SOD2 signaling pathway in hearing protection by maintaining the redox state of mitochondria in hair cells after NE (Figure 7). Our findings reveal an interaction between SIRT3 and noise-induced oxidative stress in hair cells and suggest a potential therapeutic strategy to improve NIHL by activating SIRT3-mediated SOD2 deacetylation.

## Data Availability

The original contributions presented in the study are included in the article/Supplementary Material, further inquiries can be directed to the corresponding authors.
