# Supplementary figures and images for: Sirtuin-3 Protects Cochlear Hair Cells Against Noise-Induced Damage via the Superoxide Dismutase 2/Reactive Oxygen Species Signaling Pathway
Source: Front Cell Dev Biol. 2021 Nov 18;9:766512. doi: 10.3389/fcell.2021.766512 (PMC8637754; doi:10.3389/fcell.2021.766512)

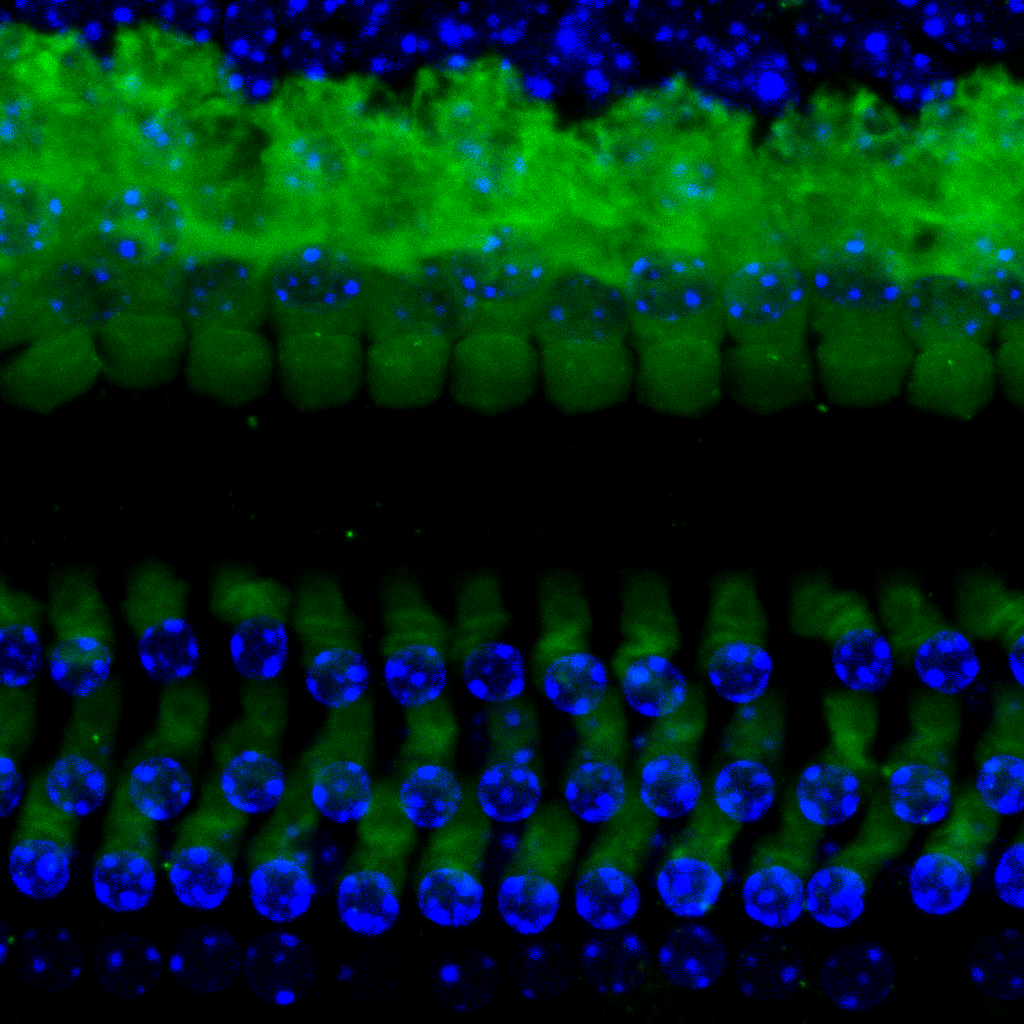

Supplement: Supplementary file 1 [file DataSheet1.ZIP › supplementary/figure2/2A/apical/CONTROL.tif]

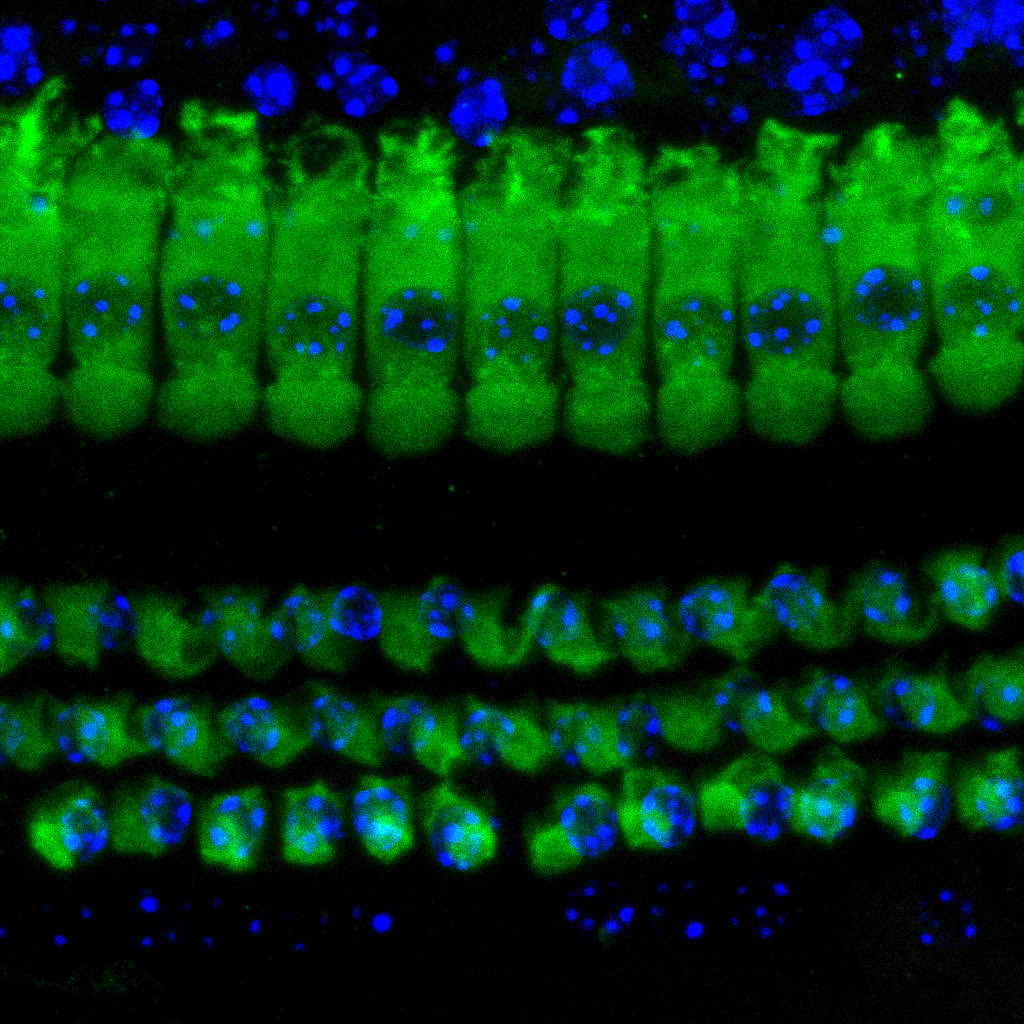

Supplement: Supplementary file 1 [file DataSheet1.ZIP › supplementary/figure2/2A/apical/PTS.tif]

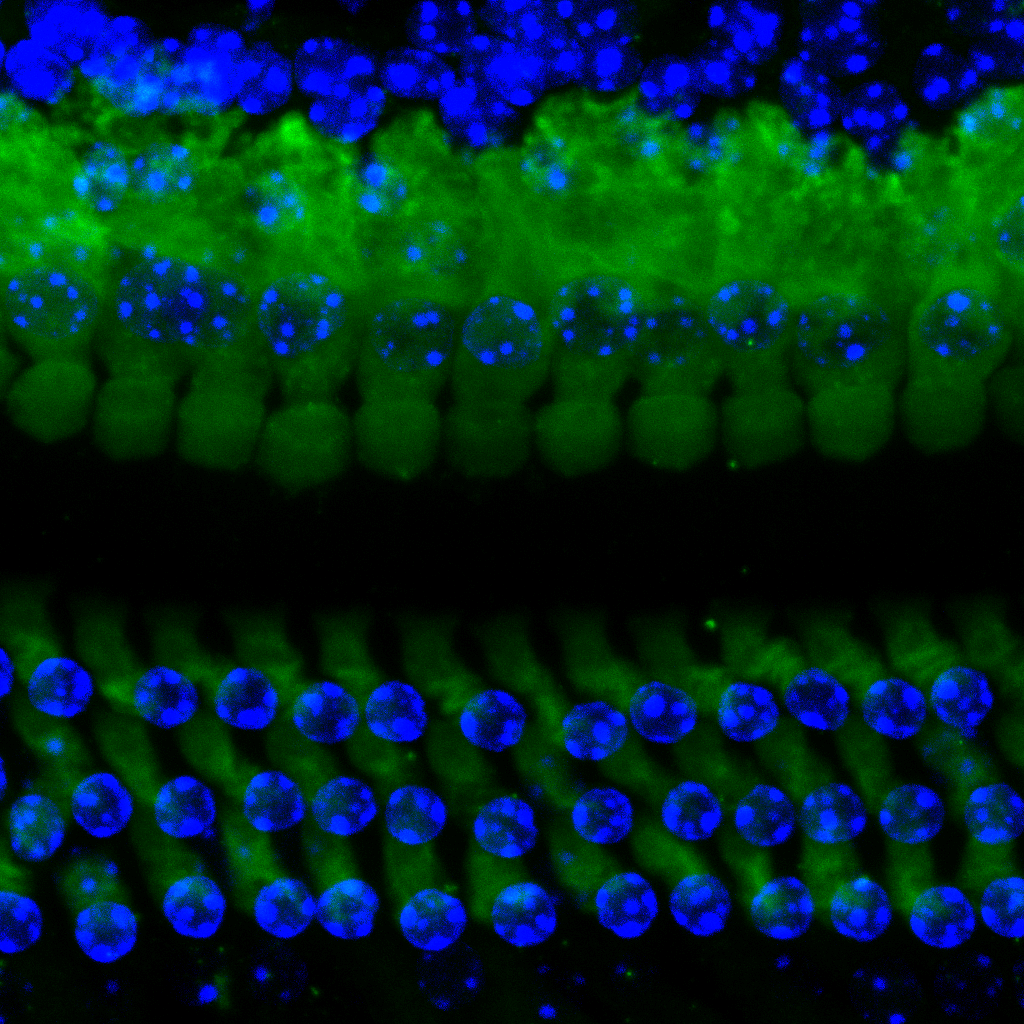

Supplement: Supplementary file 1 [file DataSheet1.ZIP › supplementary/figure2/2A/apical/TTS.tif]

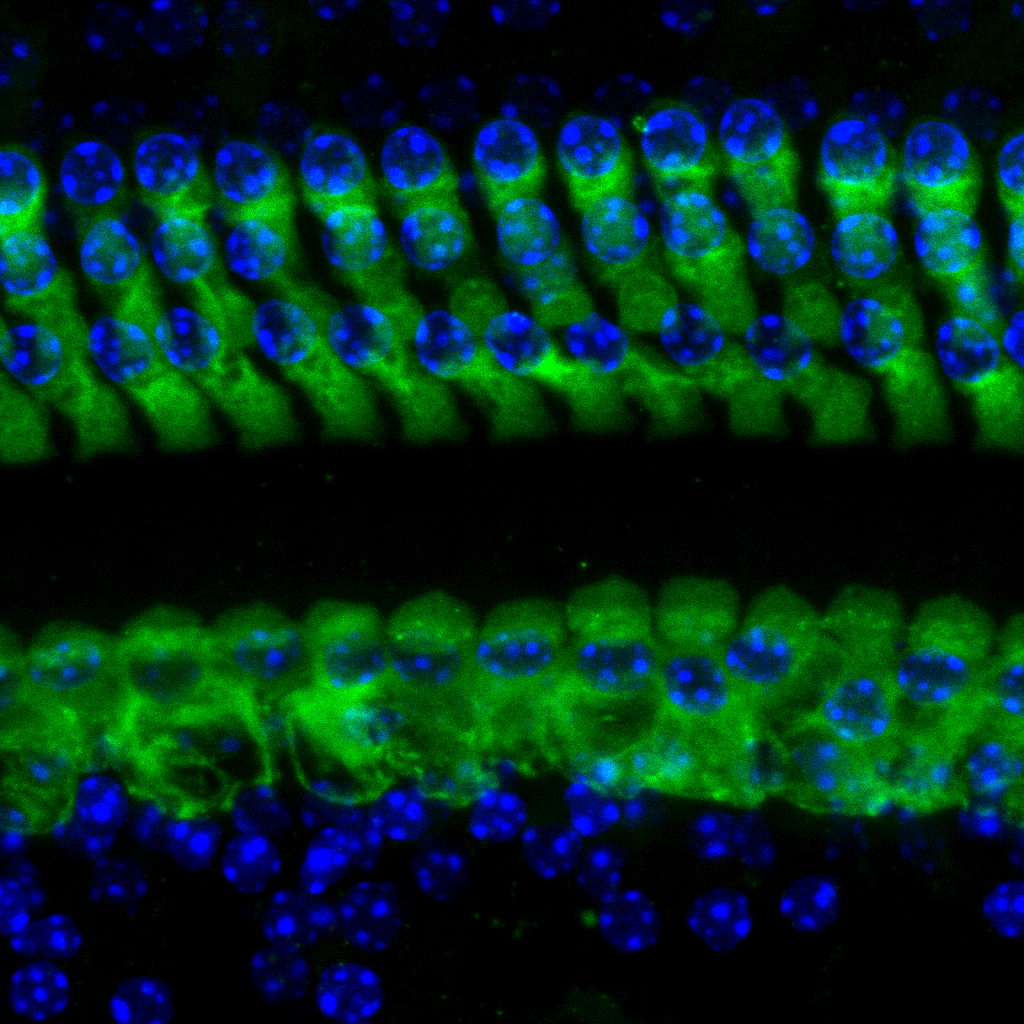

Supplement: Supplementary file 1 [file DataSheet1.ZIP › supplementary/figure2/2A/basal/CONTROL.tif]

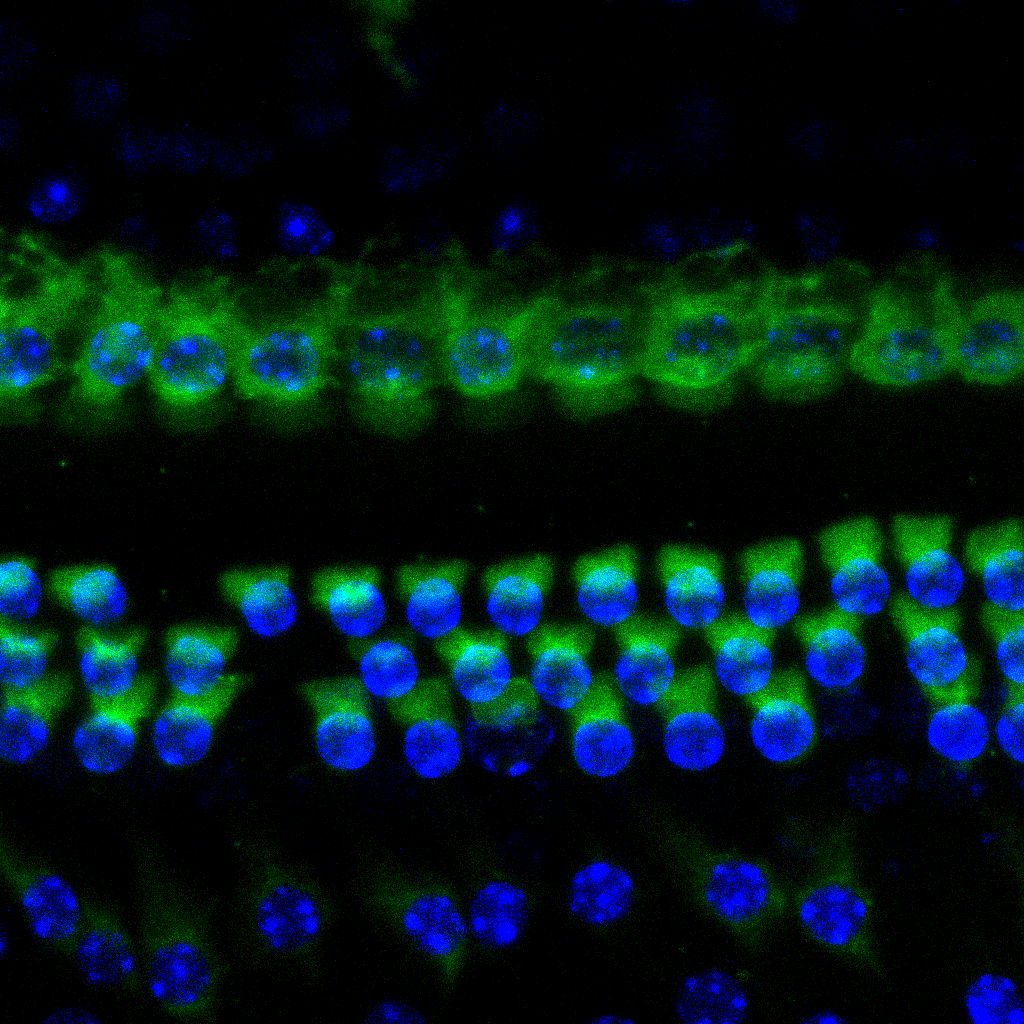

Supplement: Supplementary file 1 [file DataSheet1.ZIP › supplementary/figure2/2A/basal/PTS.tif]

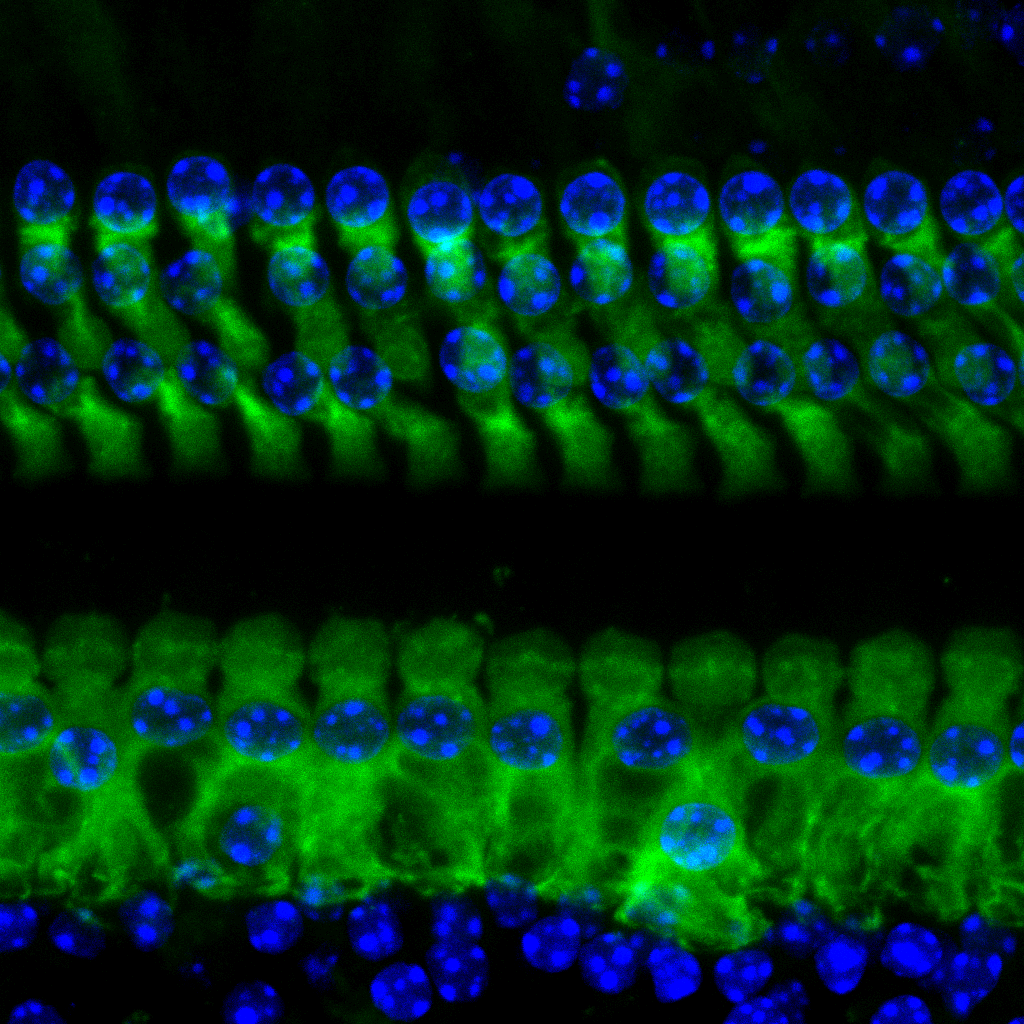

Supplement: Supplementary file 1 [file DataSheet1.ZIP › supplementary/figure2/2A/basal/TTS.tif]

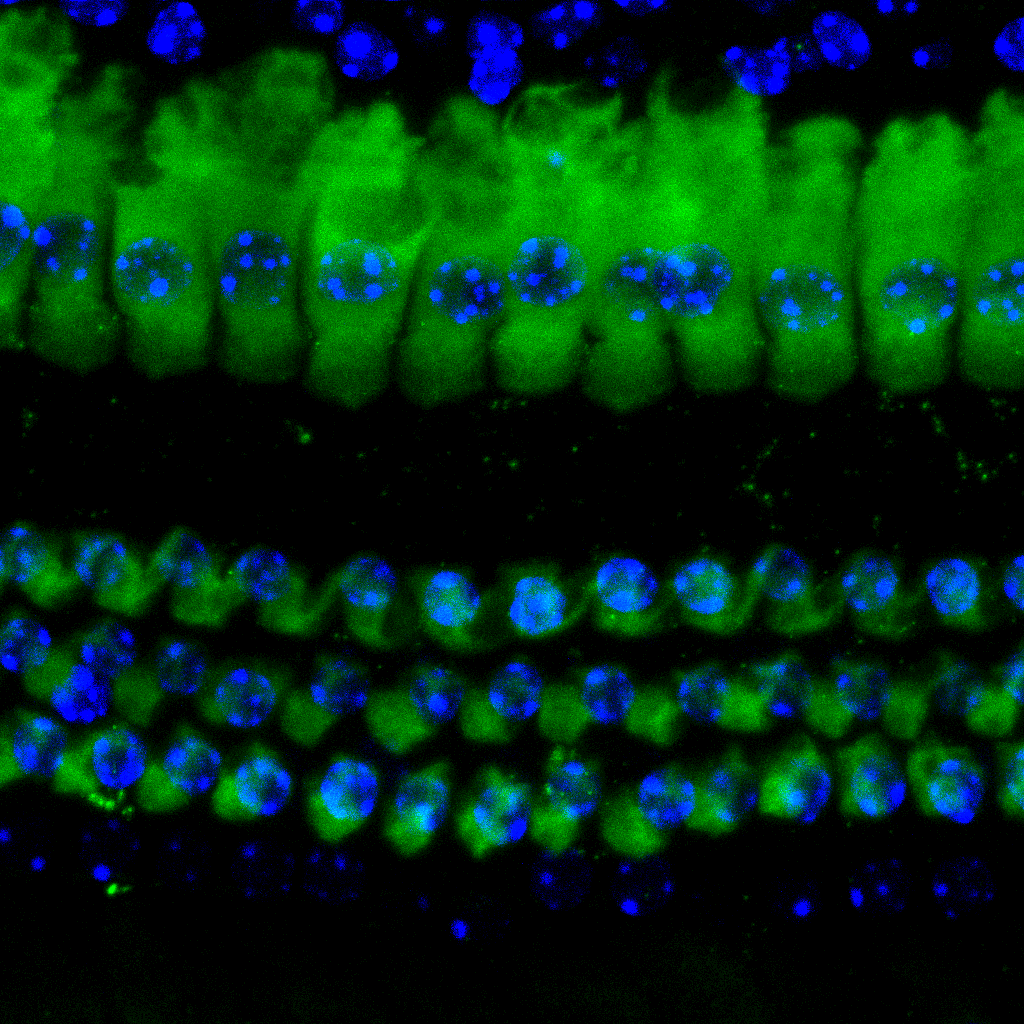

Supplement: Supplementary file 1 [file DataSheet1.ZIP › supplementary/figure2/2A/middle/CONTROL.tif]

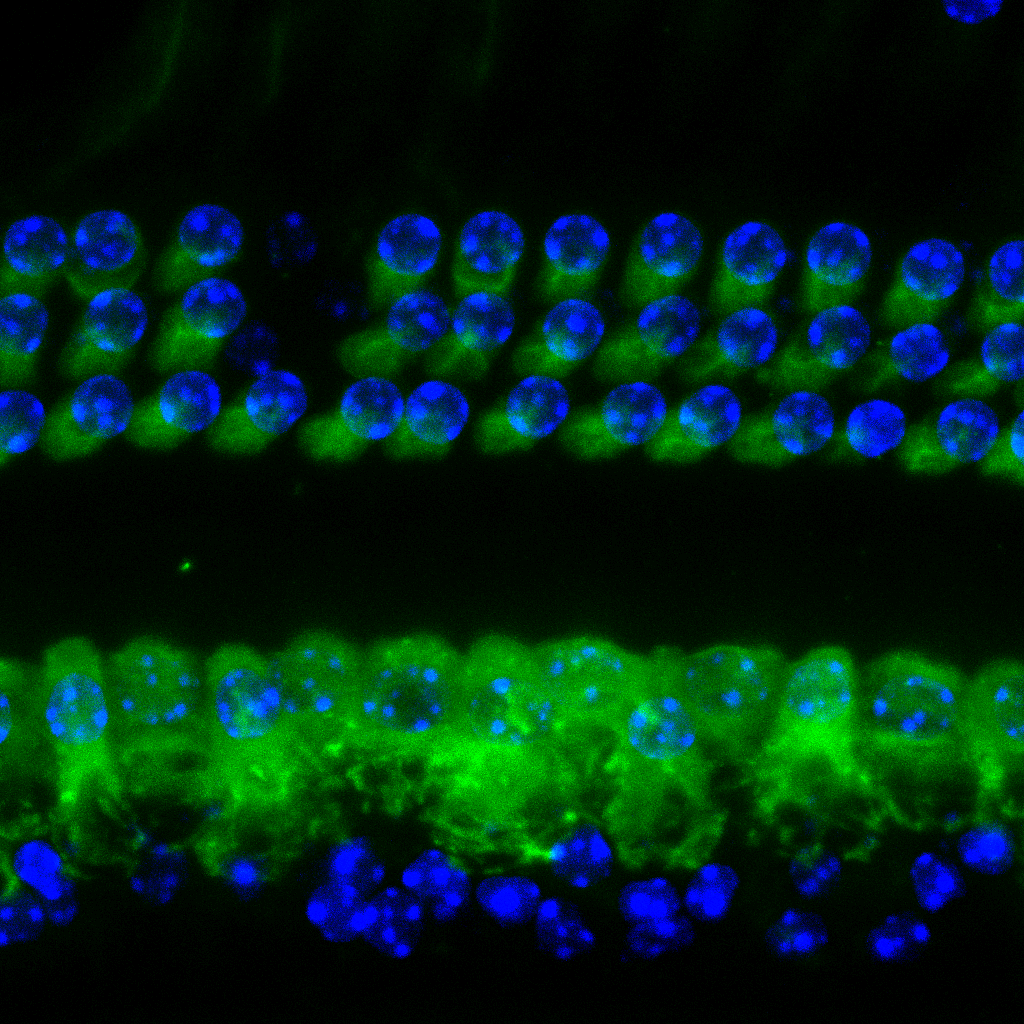

Supplement: Supplementary file 1 [file DataSheet1.ZIP › supplementary/figure2/2A/middle/PTS.tif]

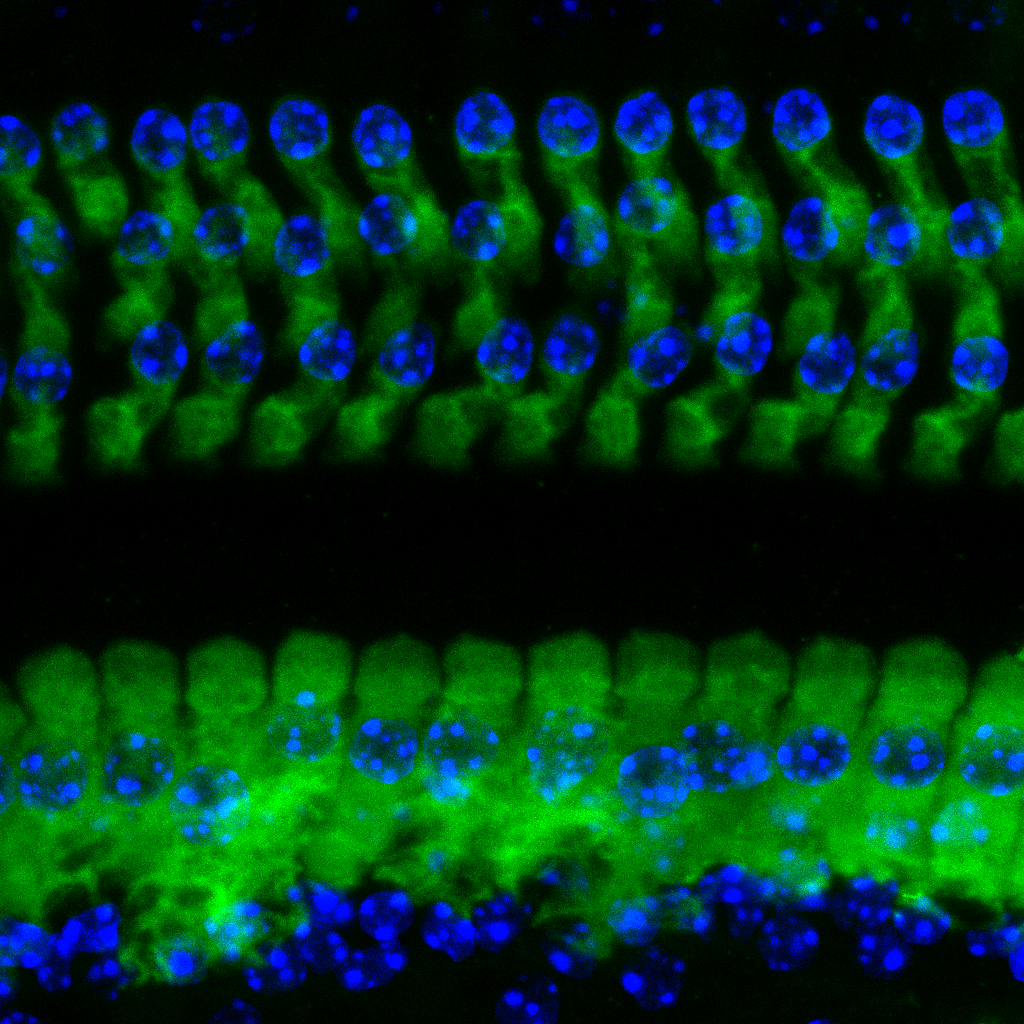

Supplement: Supplementary file 1 [file DataSheet1.ZIP › supplementary/figure2/2A/middle/TTS.tif]

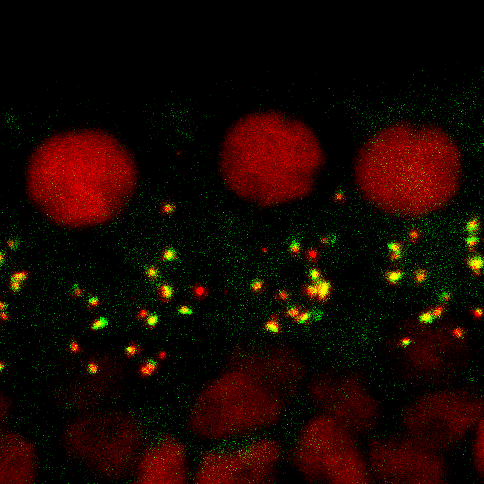

Supplement: Supplementary file 1 [file DataSheet1.ZIP › supplementary/figure2/2C/apical/CONTROL.tif]

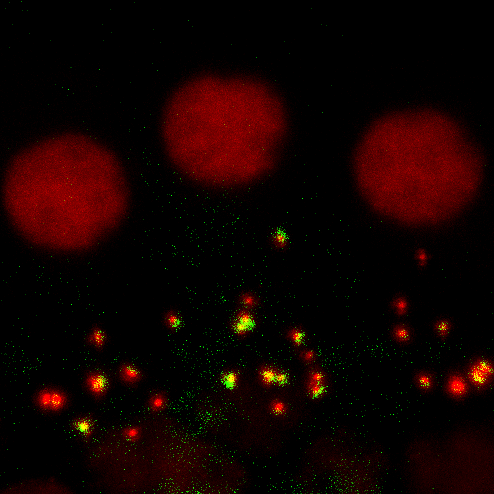

Supplement: Supplementary file 1 [file DataSheet1.ZIP › supplementary/figure2/2C/apical/PTS.tif]

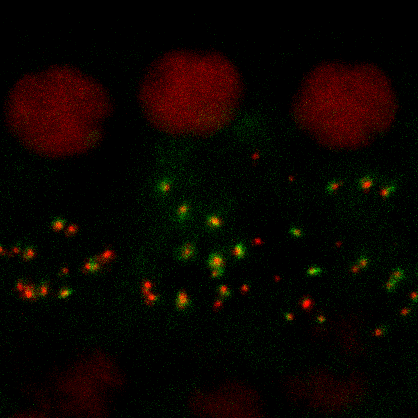

Supplement: Supplementary file 1 [file DataSheet1.ZIP › supplementary/figure2/2C/apical/TTS.tif]

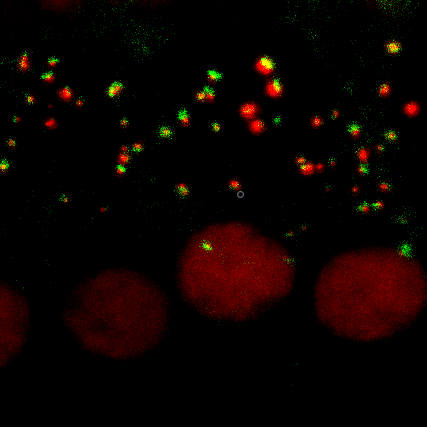

Supplement: Supplementary file 1 [file DataSheet1.ZIP › supplementary/figure2/2C/basal/control.tif]

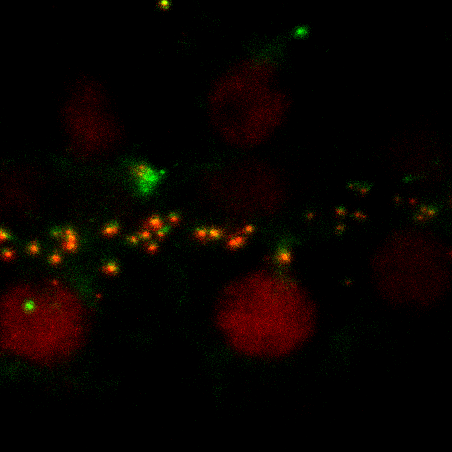

Supplement: Supplementary file 1 [file DataSheet1.ZIP › supplementary/figure2/2C/basal/PTS.tiff]

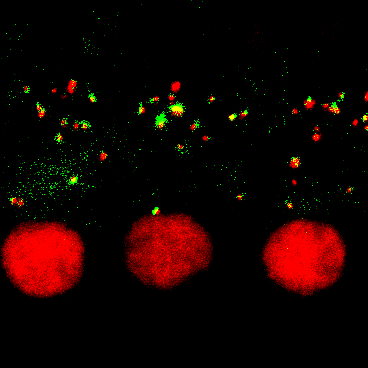

Supplement: Supplementary file 1 [file DataSheet1.ZIP › supplementary/figure2/2C/basal/TTS.tif]

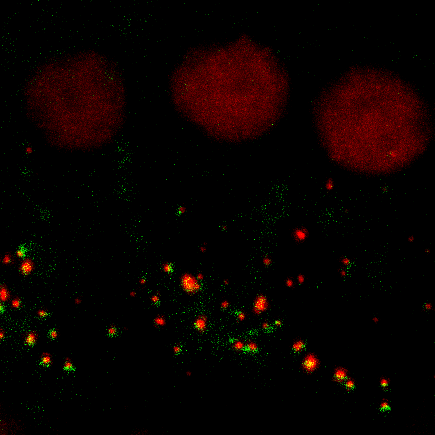

Supplement: Supplementary file 1 [file DataSheet1.ZIP › supplementary/figure2/2C/middle/control.tif]

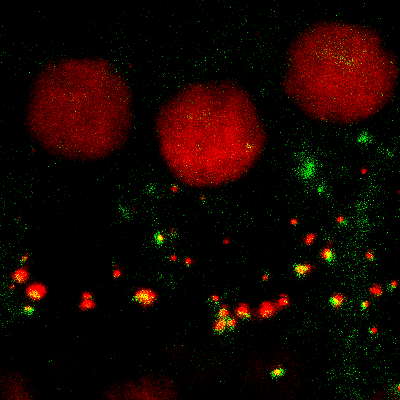

Supplement: Supplementary file 1 [file DataSheet1.ZIP › supplementary/figure2/2C/middle/pts.tif]

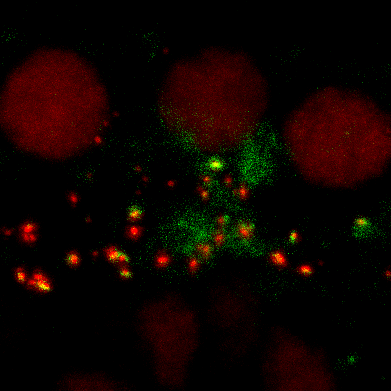

Supplement: Supplementary file 1 [file DataSheet1.ZIP › supplementary/figure2/2C/middle/TTS.tif]

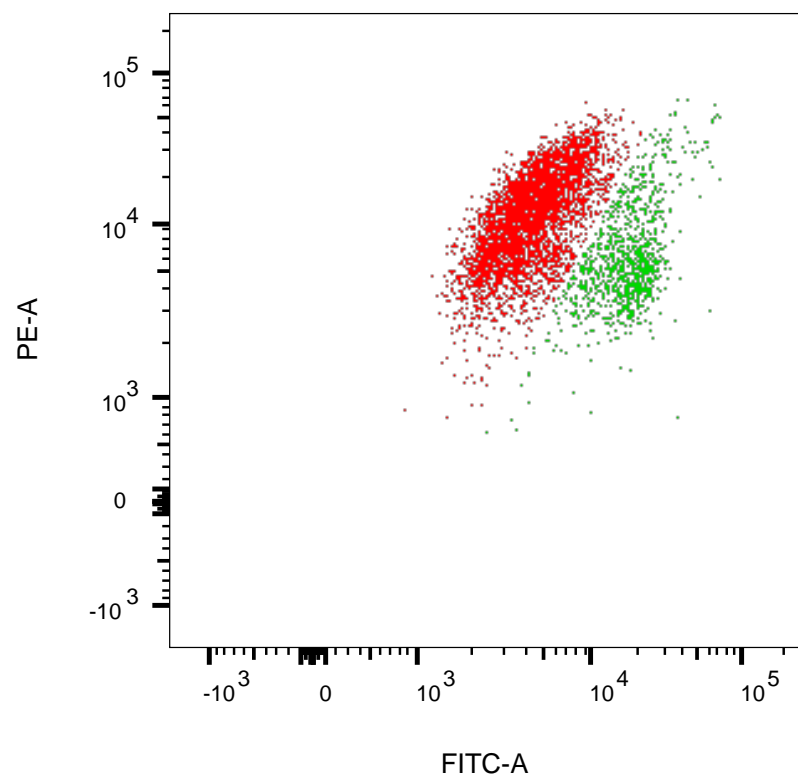

|                                                                                   | Sample Name              | Subset Name | Count |
|-----------------------------------------------------------------------------------|--------------------------|-------------|-------|
| 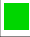 | Specimen_001_4-1_002.fcs | green       | 1003  |
| 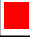 | Specimen_001_4-1_002.fcs | red         | 4447  |

Supplement: Supplementary file 1 [file DataSheet1.ZIP › supplementary/figure3/3A/control.pdf]

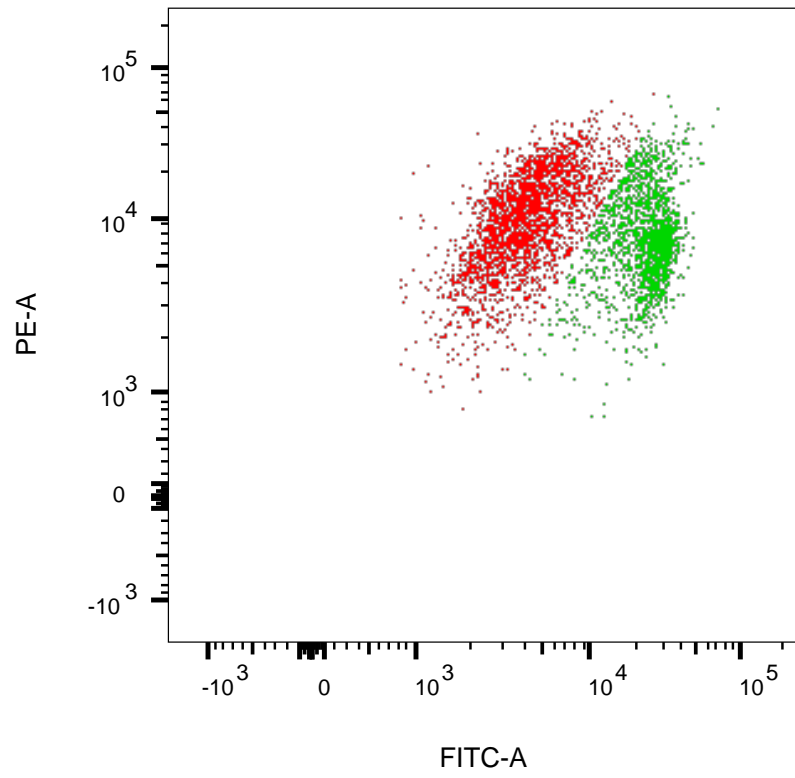

|                                                                                   | Sample Name               | Subset Name | Count |
|-----------------------------------------------------------------------------------|---------------------------|-------------|-------|
| 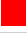 | Specimen_001_PTS2_002.fcs | red         | 2590  |
| 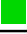 | Specimen_001_PTS2_002.fcs | green       | 1772  |

Supplement: Supplementary file 1 [file DataSheet1.ZIP › supplementary/figure3/3A/NE2.pdf]

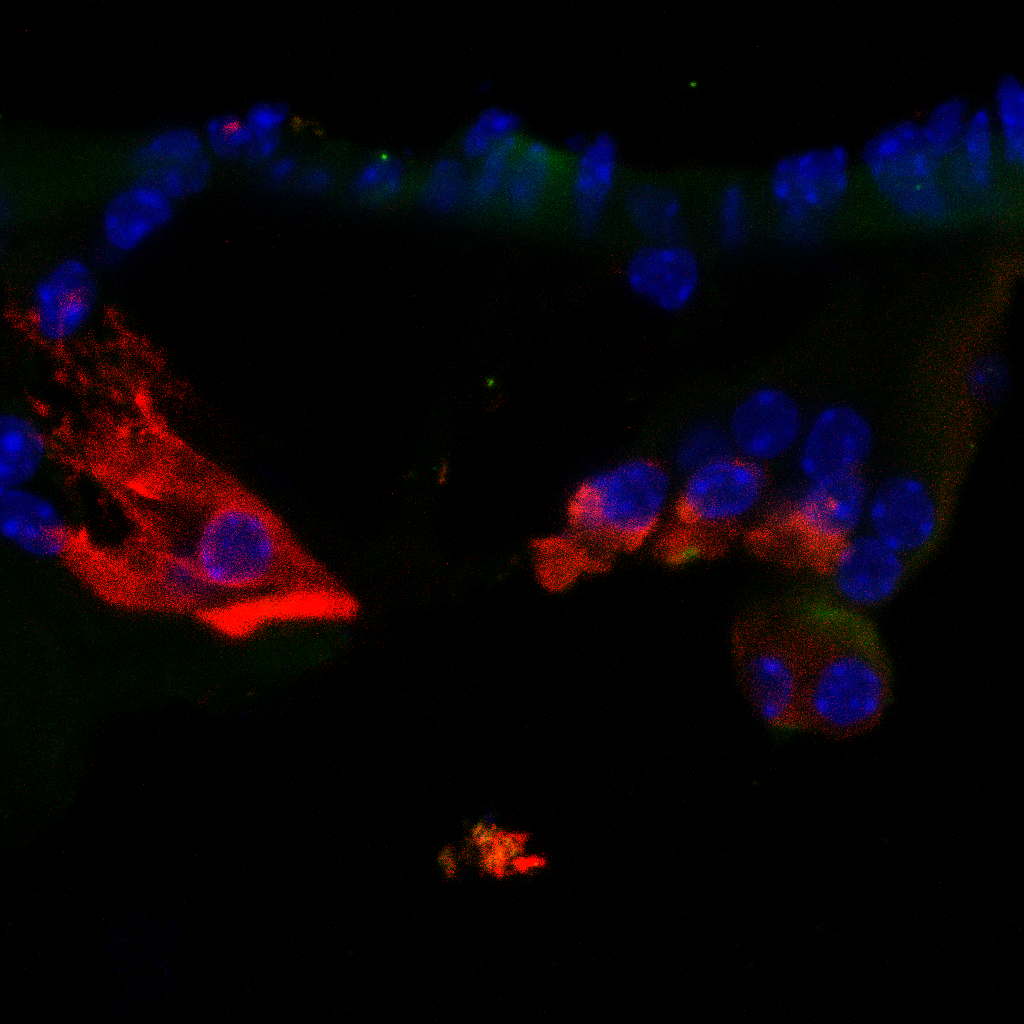

Supplement: Supplementary file 1 [file DataSheet1.ZIP › supplementary/figure3/3C/Control/Project001_Series025_Crop001_Processed001.tif]

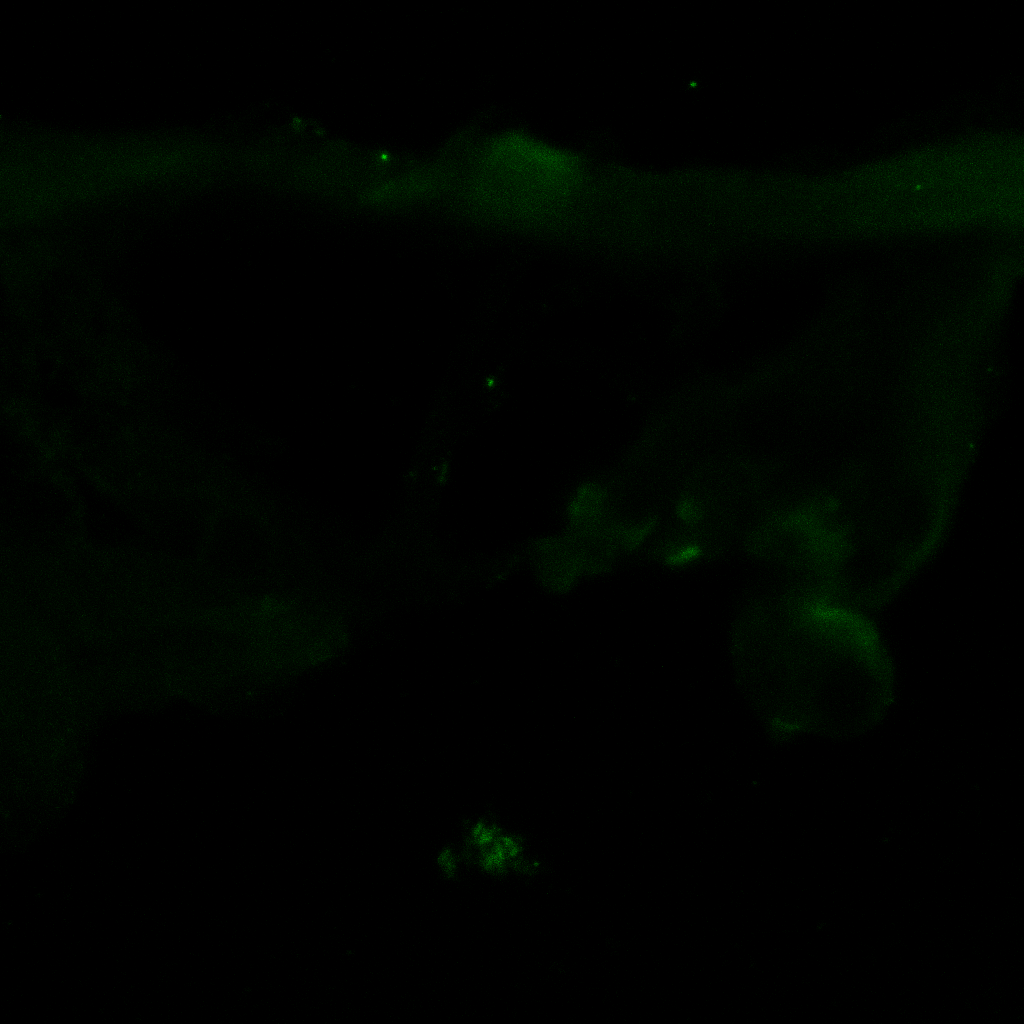

Supplement: Supplementary file 1 [file DataSheet1.ZIP › supplementary/figure3/3C/Control/Project001_Series025_Crop001_Processed001_ch00.tif]

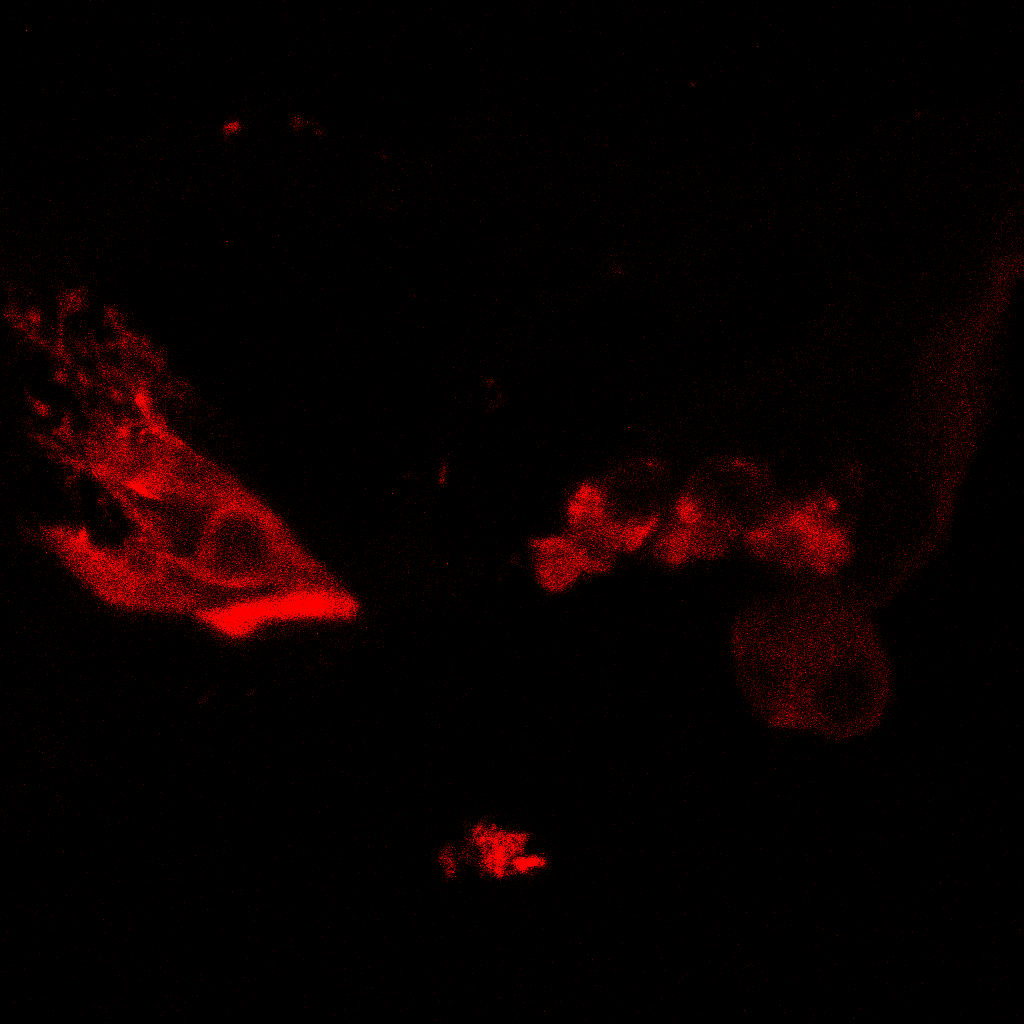

Supplement: Supplementary file 1 [file DataSheet1.ZIP › supplementary/figure3/3C/Control/Project001_Series025_Crop001_Processed001_ch01.tif]

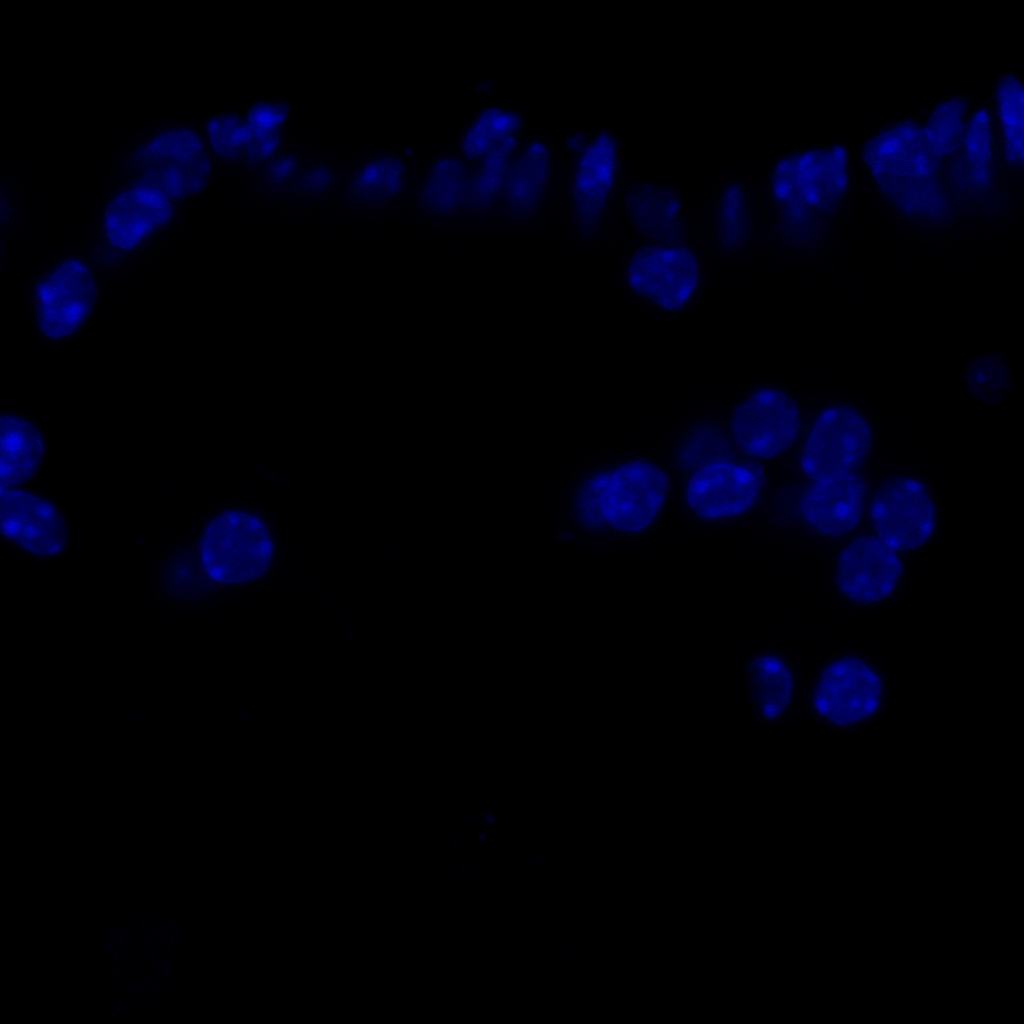

Supplement: Supplementary file 1 [file DataSheet1.ZIP › supplementary/figure3/3C/Control/Project001_Series025_Crop001_Processed001_ch02.tif]

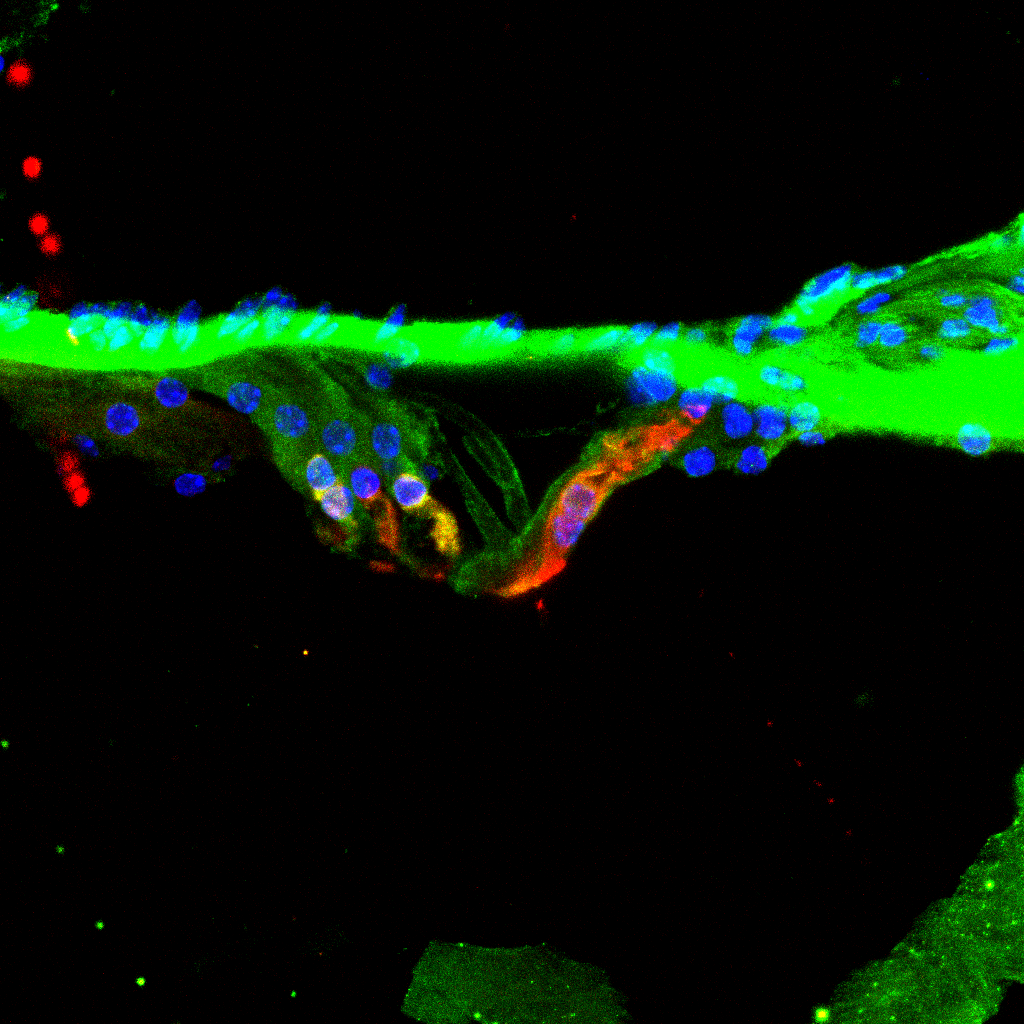

Supplement: Supplementary file 1 [file DataSheet1.ZIP › supplementary/figure3/3C/PTS/5_Series005_Processed001.tif]

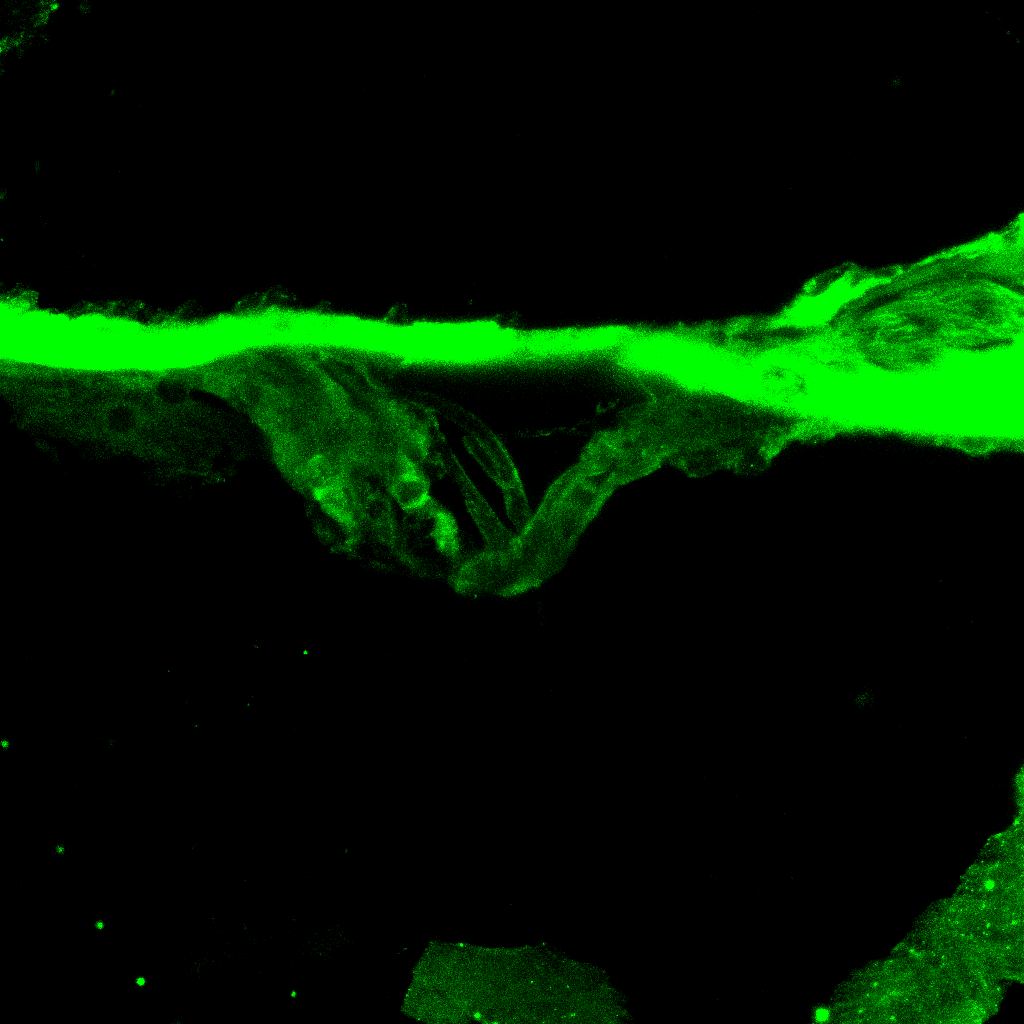

Supplement: Supplementary file 1 [file DataSheet1.ZIP › supplementary/figure3/3C/PTS/5_Series005_Processed001_ch00.tif]

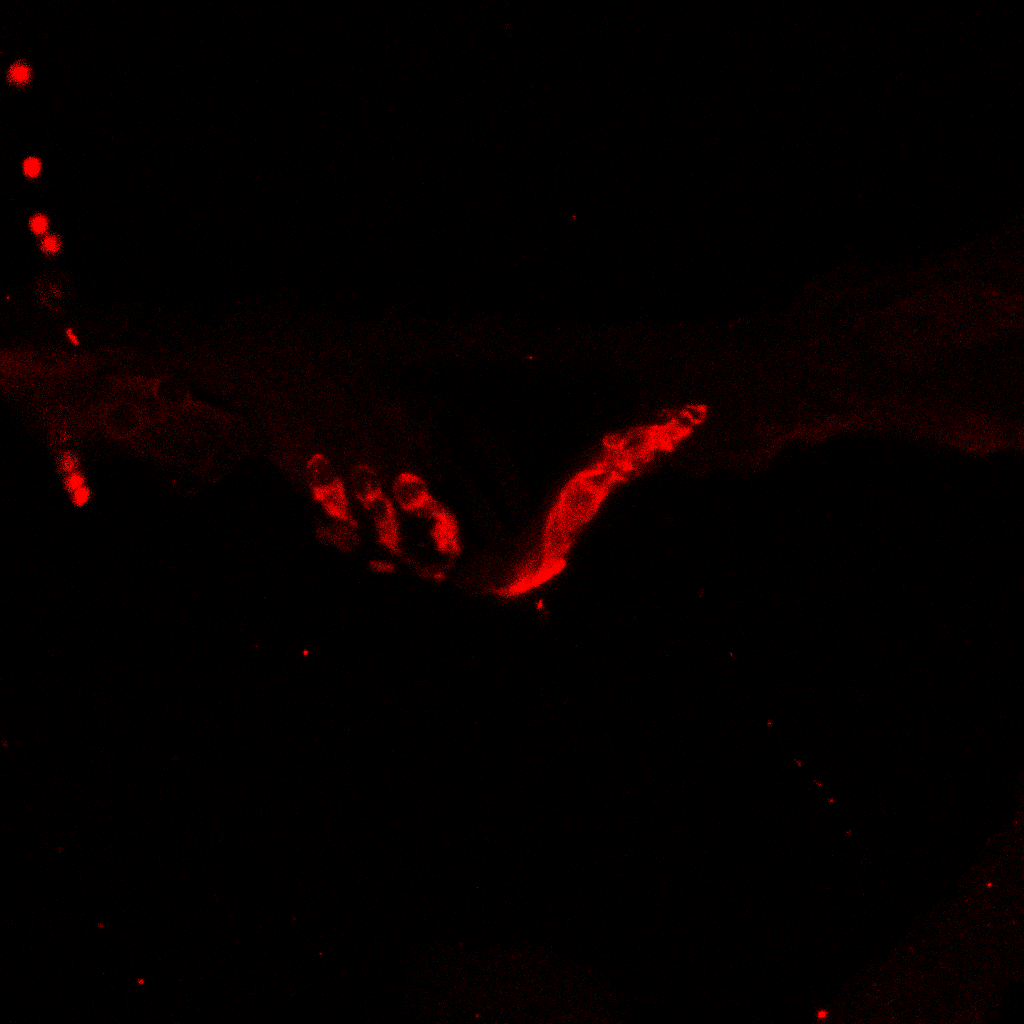

Supplement: Supplementary file 1 [file DataSheet1.ZIP › supplementary/figure3/3C/PTS/5_Series005_Processed001_ch01.tif]

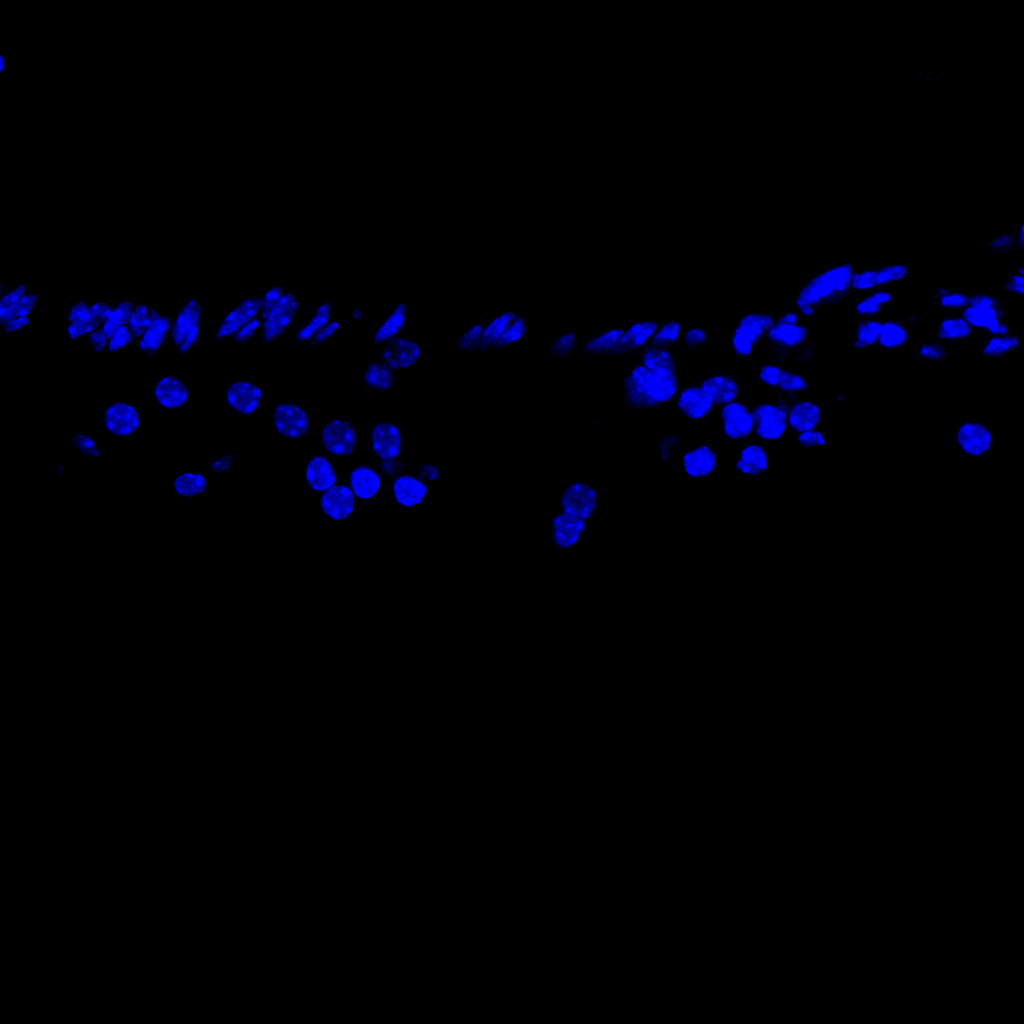

Supplement: Supplementary file 1 [file DataSheet1.ZIP › supplementary/figure3/3C/PTS/5_Series005_Processed001_ch02.tif]

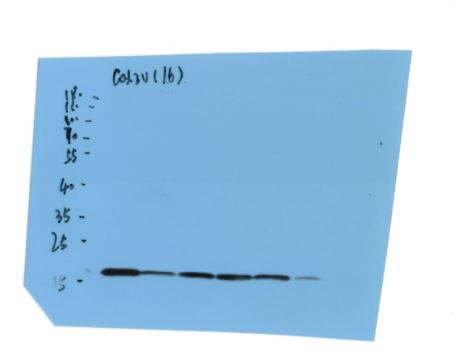

Supplement: Supplementary file 1 [file DataSheet1.ZIP › supplementary/figure3/3E/coxiv.JPG]

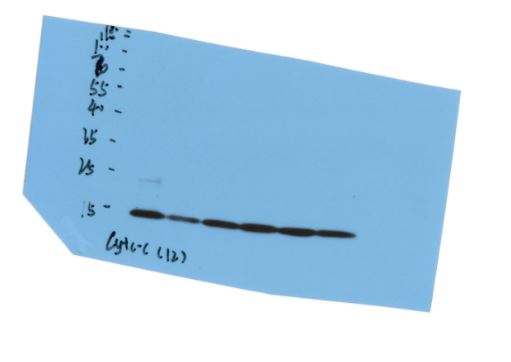

Supplement: Supplementary file 1 [file DataSheet1.ZIP › supplementary/figure3/3E/cytoc.JPG]

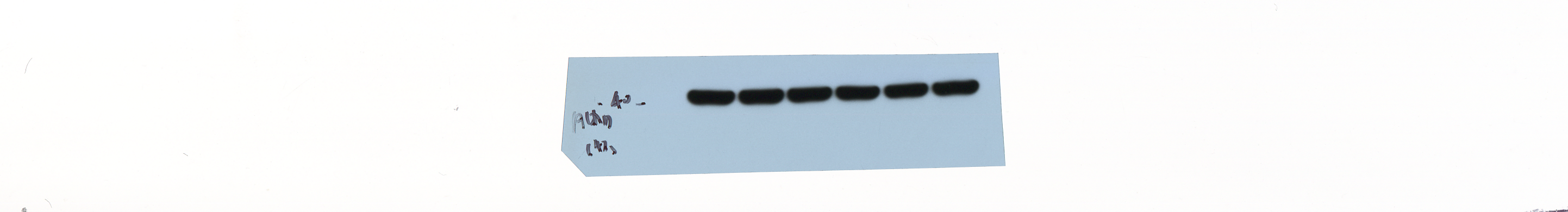

Supplement: Supplementary file 1 [file DataSheet1.ZIP › supplementary/figure3/3F/actin.tif]

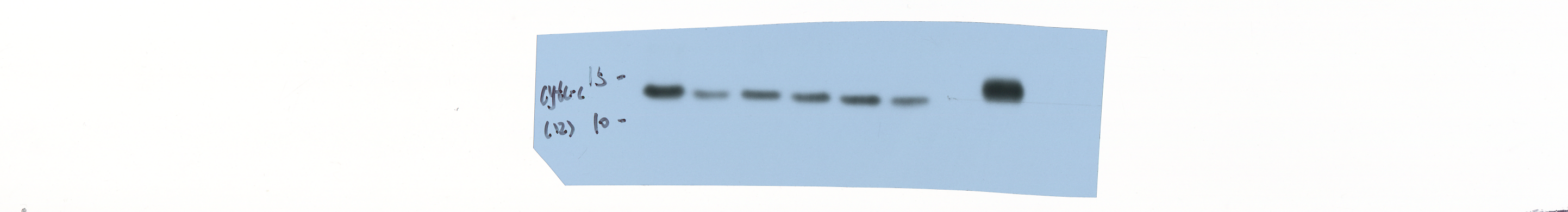

Supplement: Supplementary file 1 [file DataSheet1.ZIP › supplementary/figure3/3F/cytoc.tif]

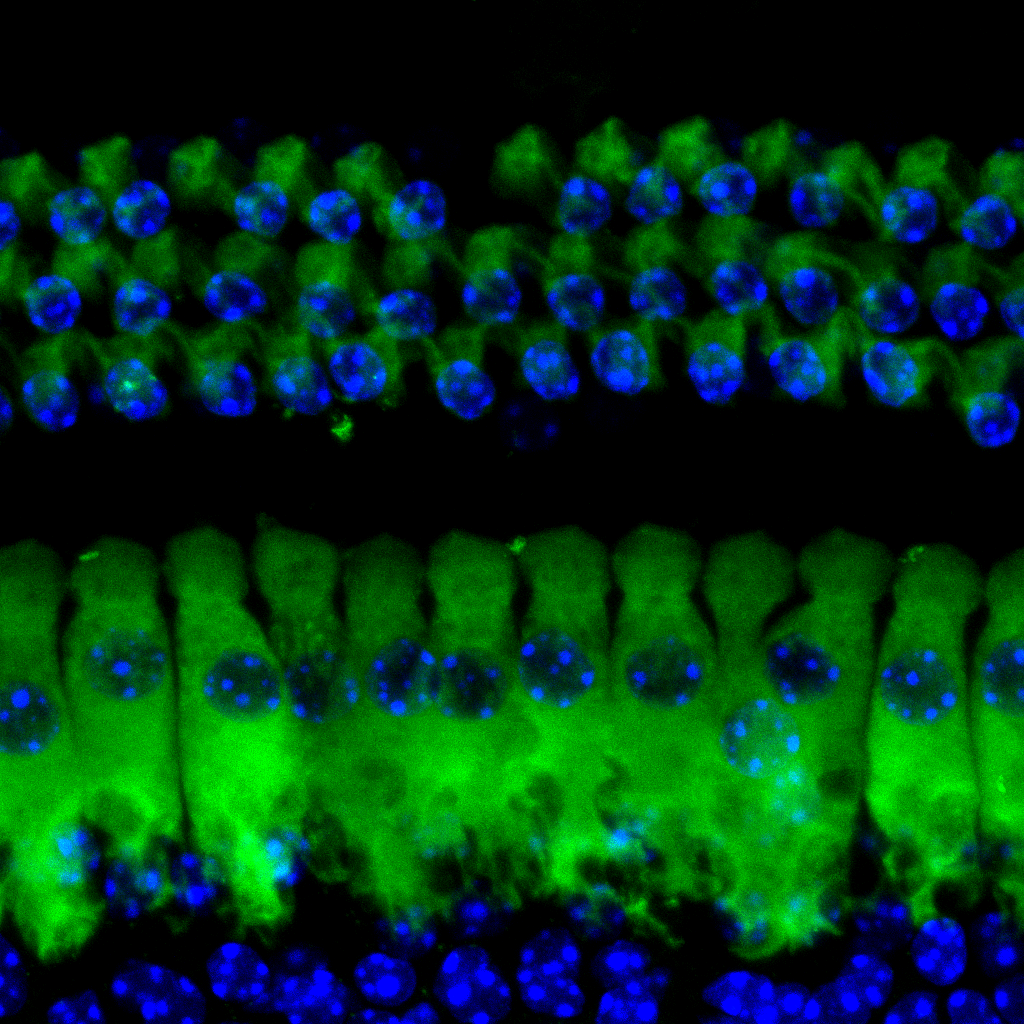

Supplement: Supplementary file 1 [file DataSheet1.ZIP › supplementary/figure4/4C/apical/PTS+3-TYP.tif]

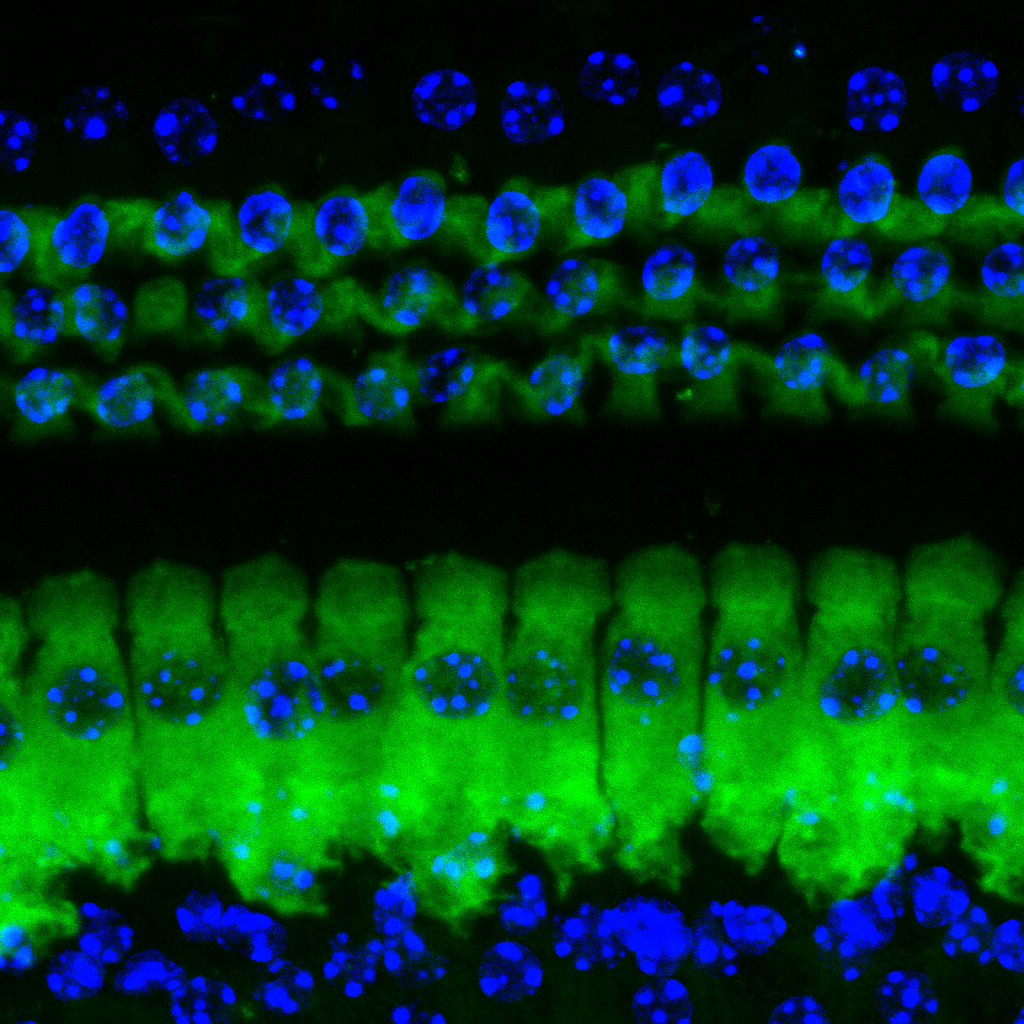

Supplement: Supplementary file 1 [file DataSheet1.ZIP › supplementary/figure4/4C/apical/PTS.tif]

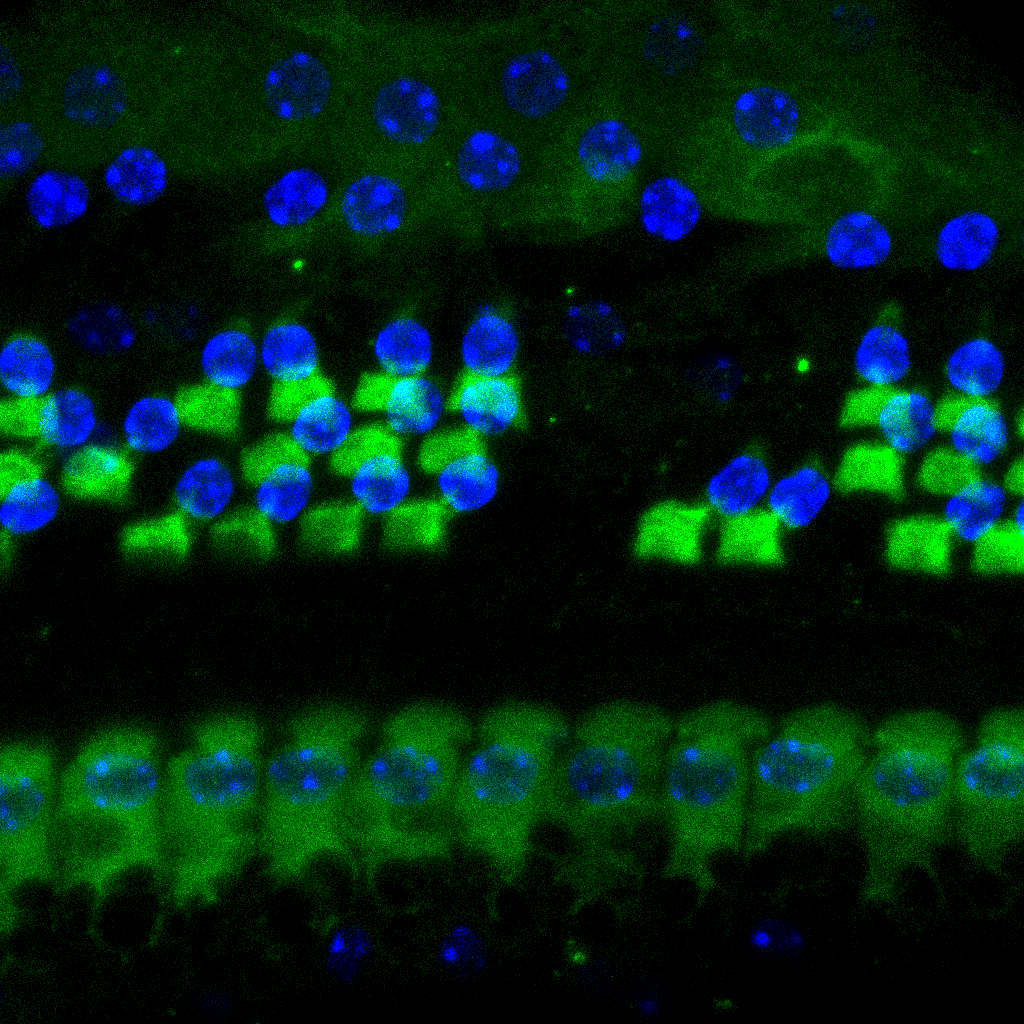

Supplement: Supplementary file 1 [file DataSheet1.ZIP › supplementary/figure4/4C/basal/PTS+3TYP.tif]

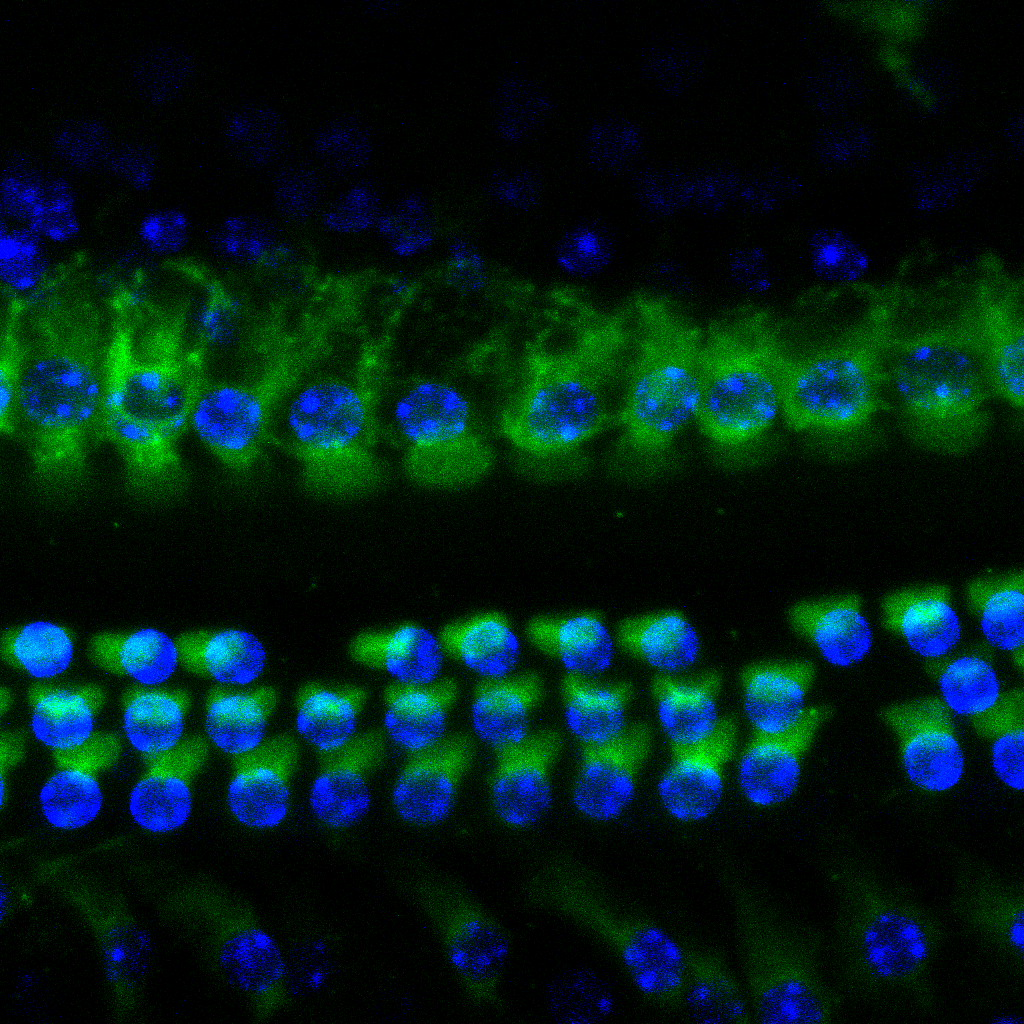

Supplement: Supplementary file 1 [file DataSheet1.ZIP › supplementary/figure4/4C/basal/PTS.tif]

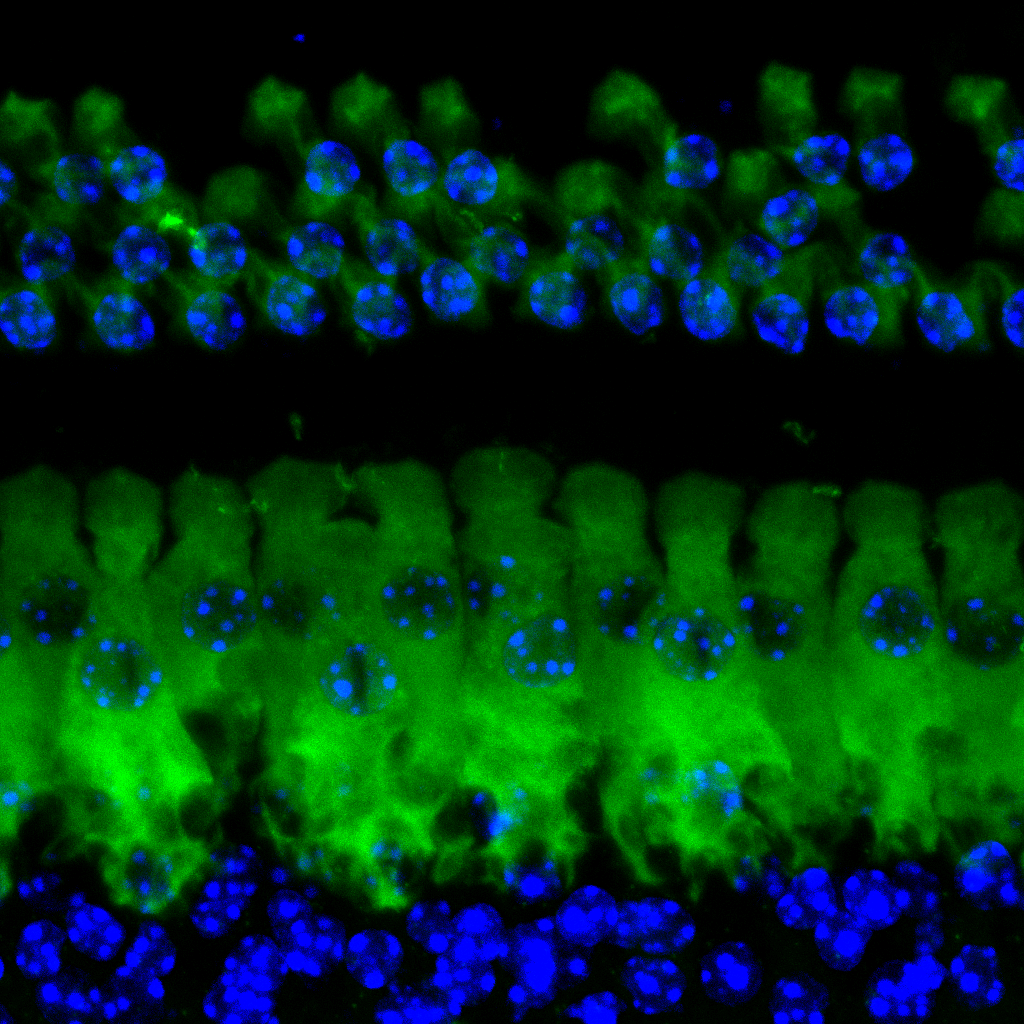

Supplement: Supplementary file 1 [file DataSheet1.ZIP › supplementary/figure4/4C/middle/PTS+3TYP.tif]

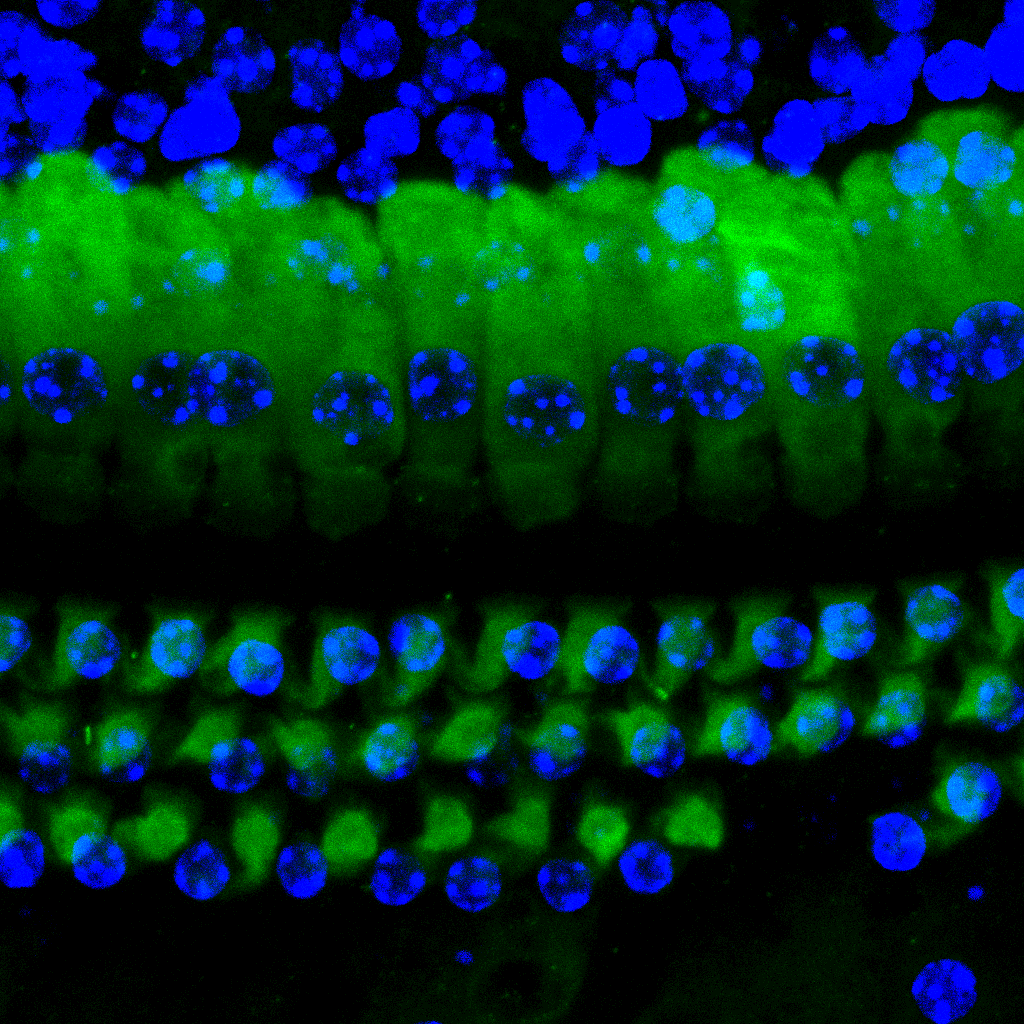

Supplement: Supplementary file 1 [file DataSheet1.ZIP › supplementary/figure4/4C/middle/PTS.tif]

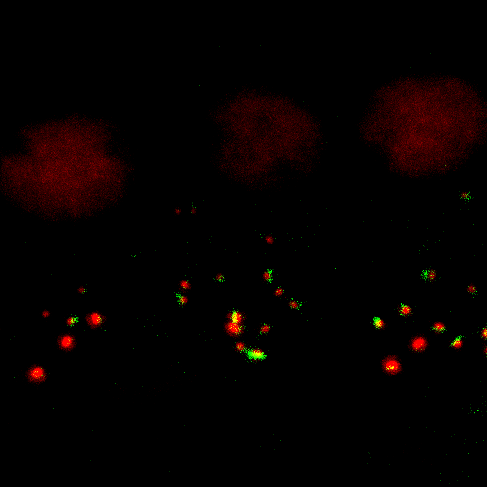

Supplement: Supplementary file 1 [file DataSheet1.ZIP › supplementary/figure4/4E/apical/PTS+3TYP.tif]

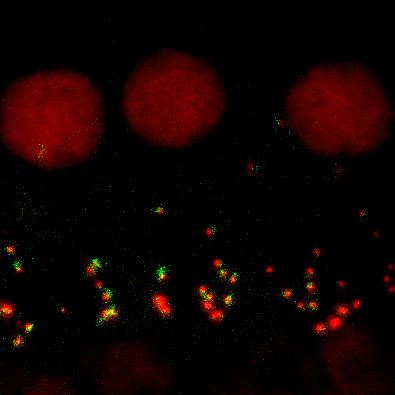

Supplement: Supplementary file 1 [file DataSheet1.ZIP › supplementary/figure4/4E/apical/PTS.tif]

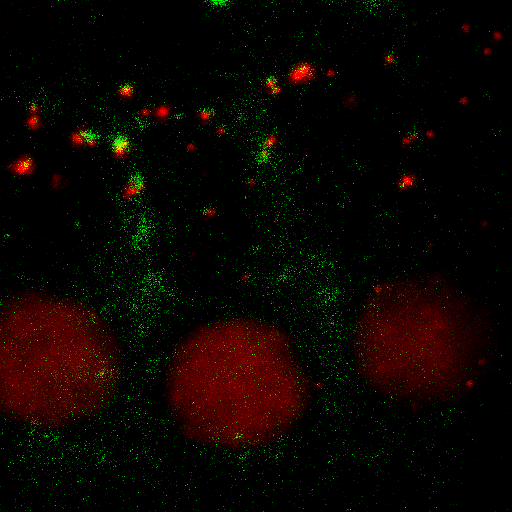

Supplement: Supplementary file 1 [file DataSheet1.ZIP › supplementary/figure4/4E/basal/PTS+3TYP.tif]

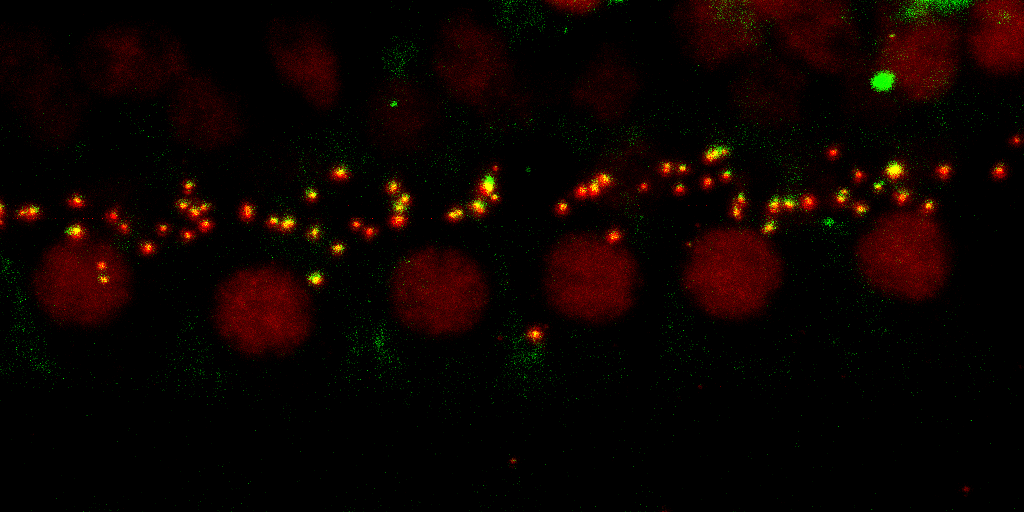

Supplement: Supplementary file 1 [file DataSheet1.ZIP › supplementary/figure4/4E/basal/PTS1.tif]

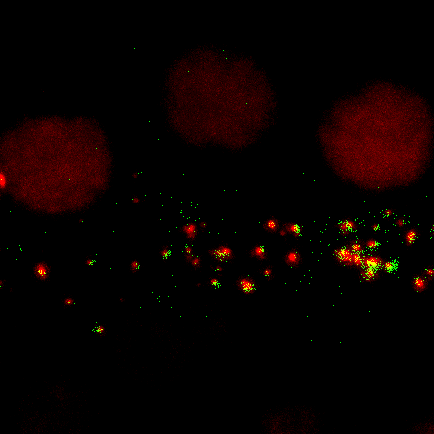

Supplement: Supplementary file 1 [file DataSheet1.ZIP › supplementary/figure4/4E/middle/PTS+3TYP.tif]

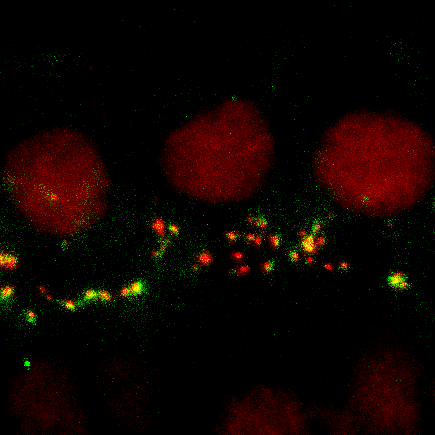

Supplement: Supplementary file 1 [file DataSheet1.ZIP › supplementary/figure4/4E/middle/PTS.tif]

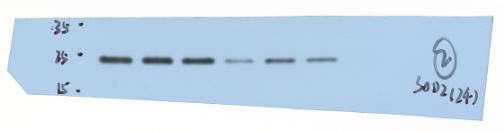

Supplement: Supplementary file 1 [file DataSheet1.ZIP › supplementary/figure5/5A/ACSOD2.JPG]

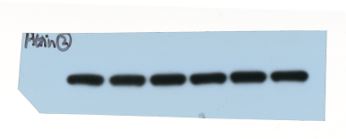

Supplement: Supplementary file 1 [file DataSheet1.ZIP › supplementary/figure5/5A/ACTIN.JPG]

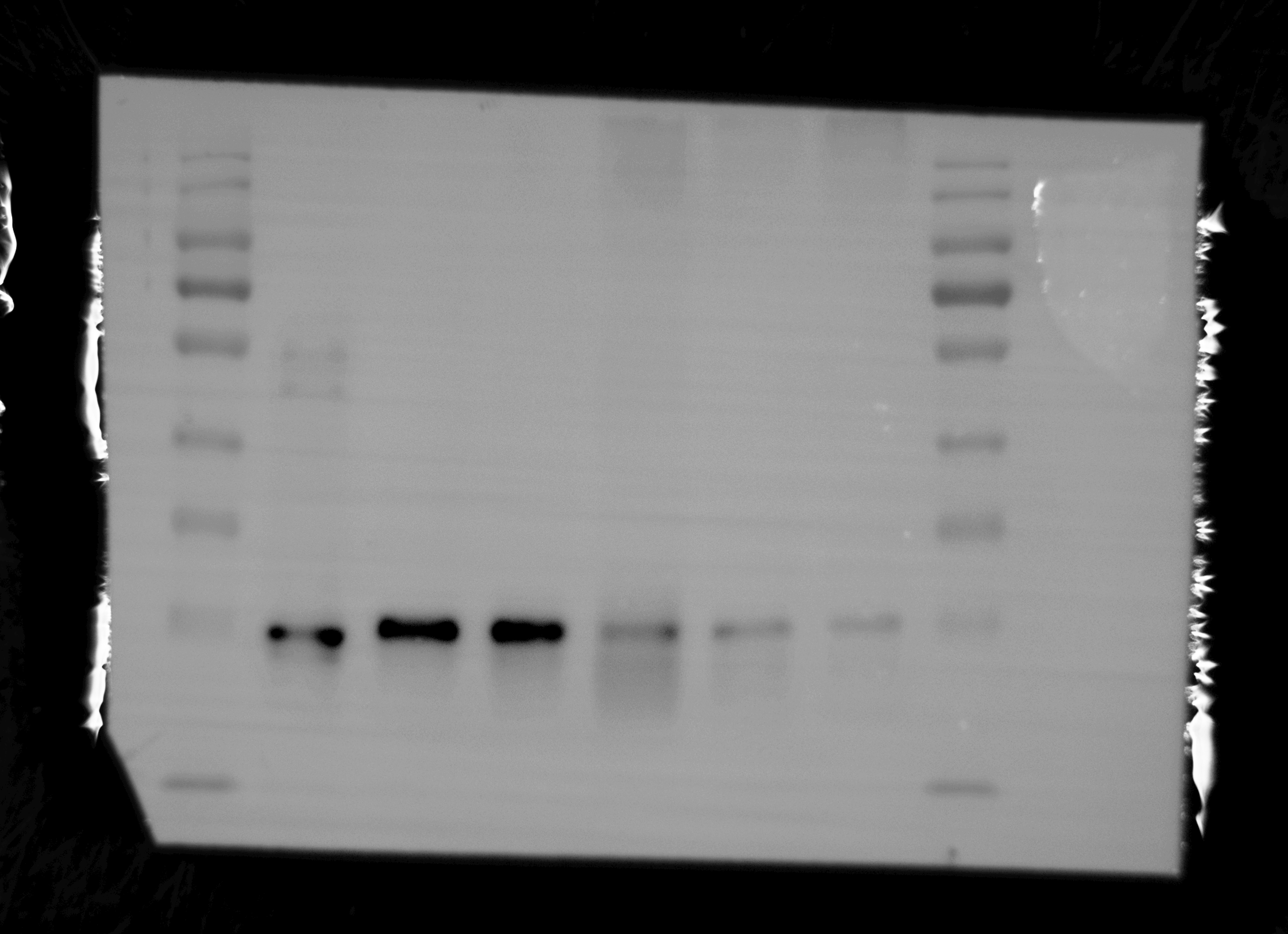

Supplement: Supplementary file 1 [file DataSheet1.ZIP › supplementary/figure5/5A/sod2.tif]

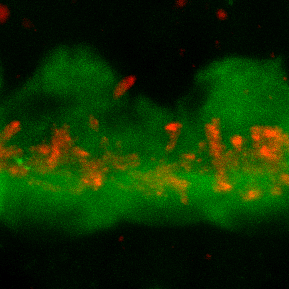

Supplement: Supplementary file 1 [file DataSheet1.ZIP › supplementary/figure5/5C/PTS/6.9 TUNEL-4HNE-RMYO-APEX_Series002_Processed001.tif]

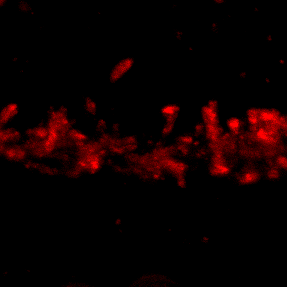

Supplement: Supplementary file 1 [file DataSheet1.ZIP › supplementary/figure5/5C/PTS/6.9 TUNEL-4HNE-RMYO-APEX_Series002_Processed001_ch02.tif]

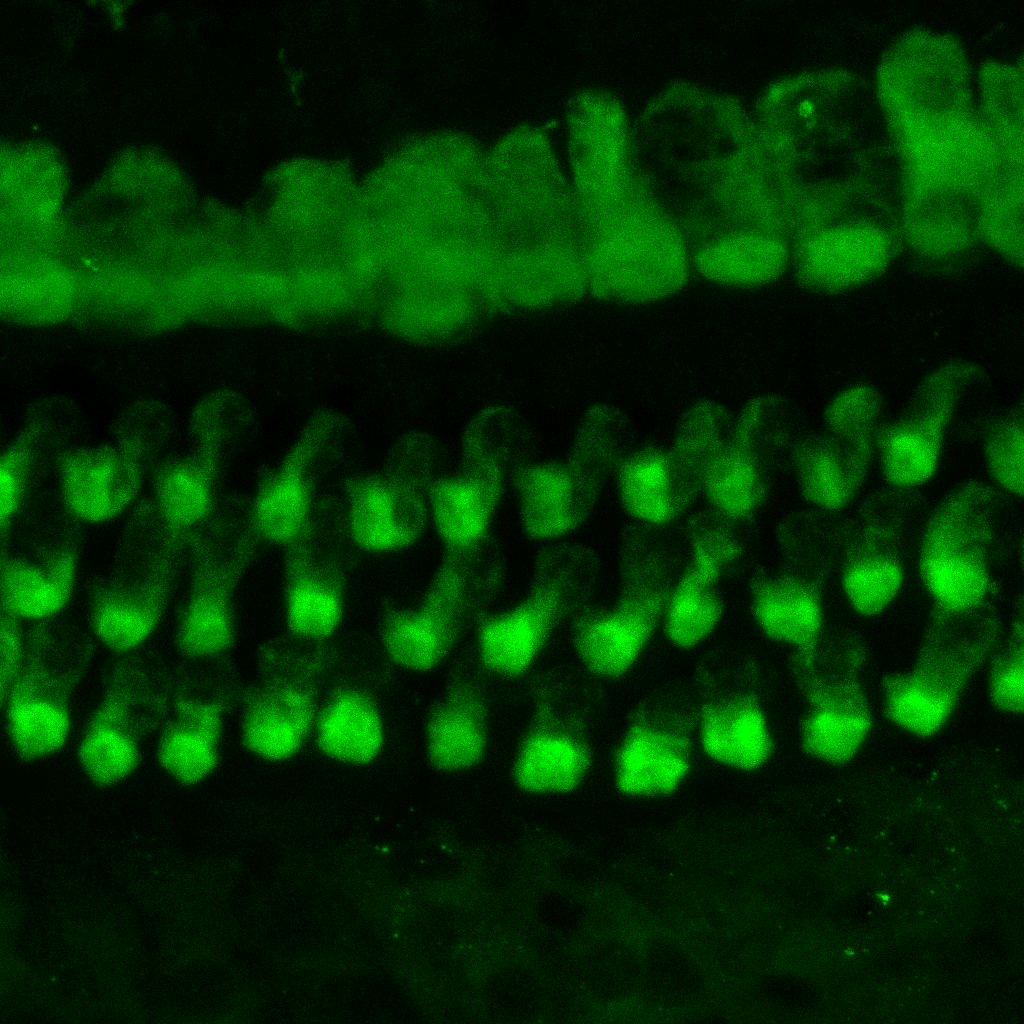

Supplement: Supplementary file 1 [file DataSheet1.ZIP › supplementary/figure5/5C/PTS/6.9 TUNEL-4HNE-RMYO-APEX_Series002_Processed001_ch03.tif]

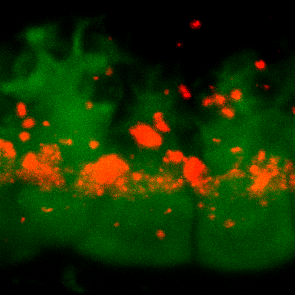

Supplement: Supplementary file 1 [file DataSheet1.ZIP › supplementary/figure5/5C/PTS+3TYP/Project001_Series003_Processed001.tif]

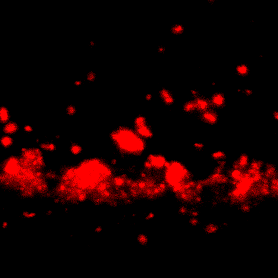

Supplement: Supplementary file 1 [file DataSheet1.ZIP › supplementary/figure5/5C/PTS+3TYP/Project001_Series003_Processed001_ch02.tif]

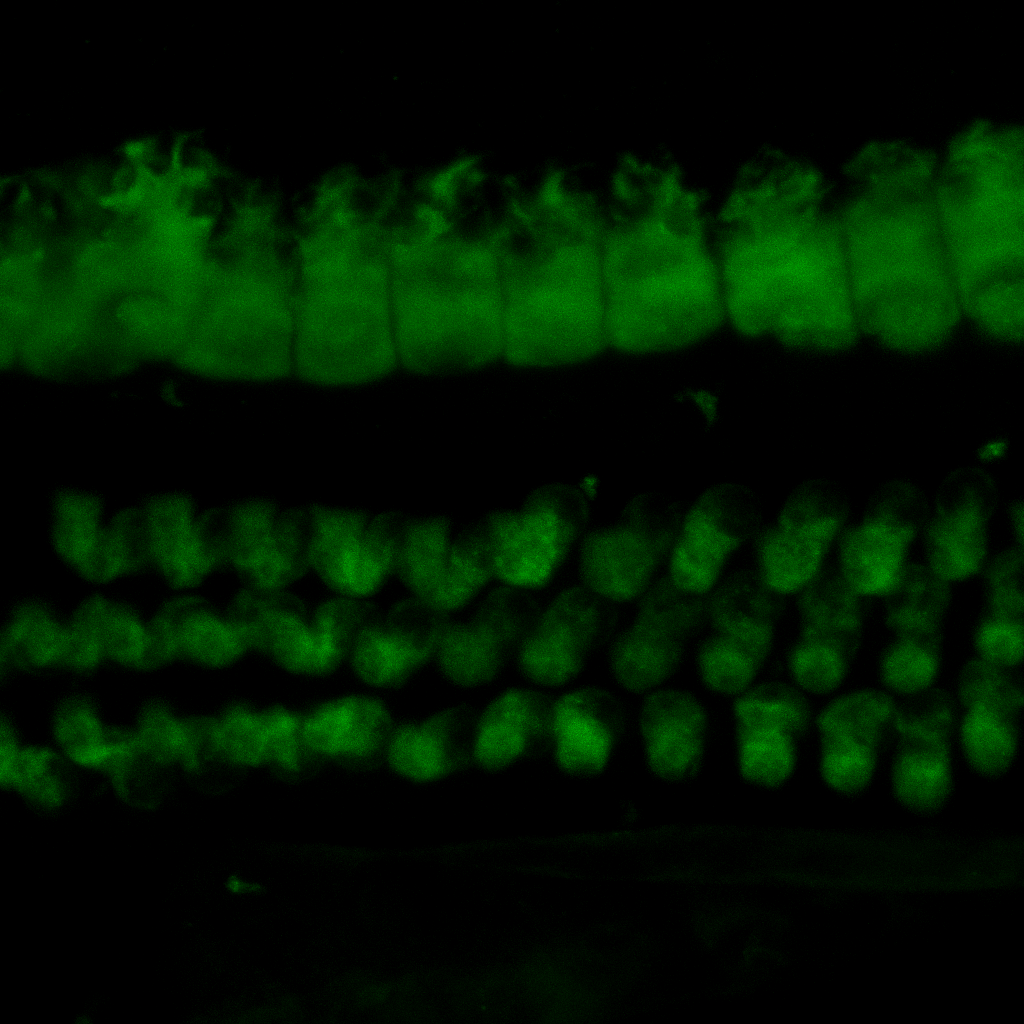

Supplement: Supplementary file 1 [file DataSheet1.ZIP › supplementary/figure5/5C/PTS+3TYP/Project001_Series003_Processed001_ch03.tif]

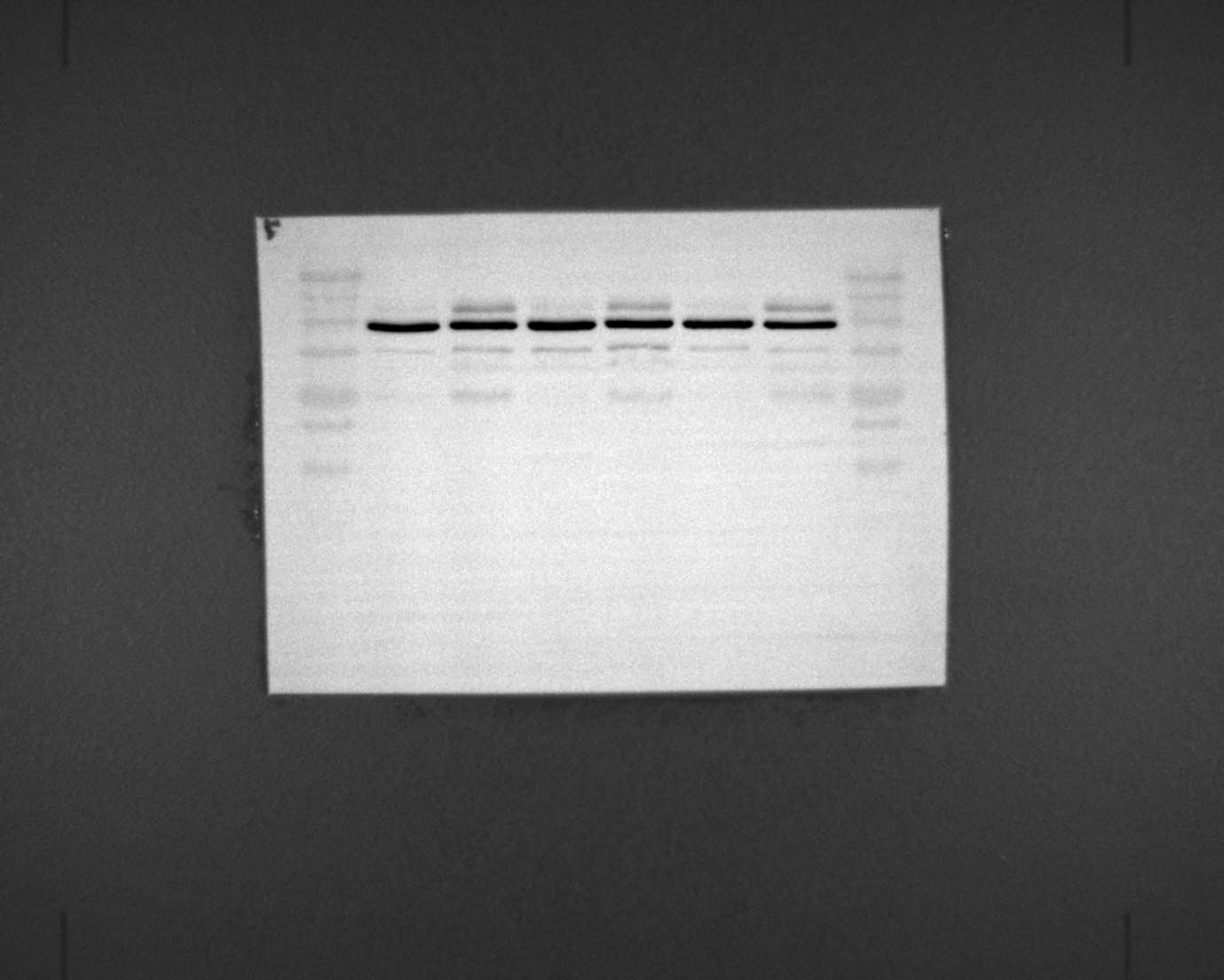

Supplement: Supplementary file 1 [file DataSheet1.ZIP › supplementary/figure5/5F/B-A M.tif]

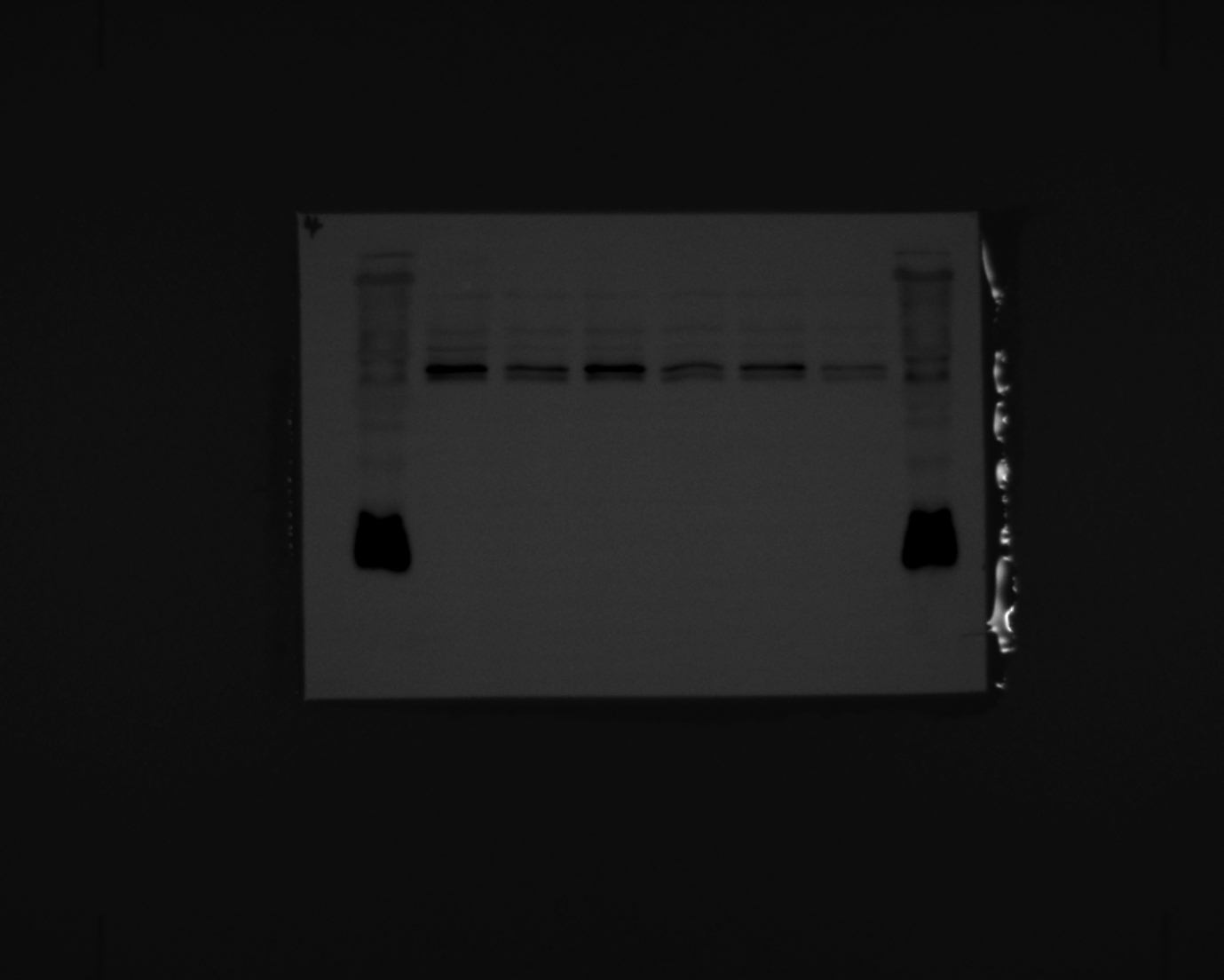

Supplement: Supplementary file 1 [file DataSheet1.ZIP › supplementary/figure5/5F/CASPASE3 M.jpg]

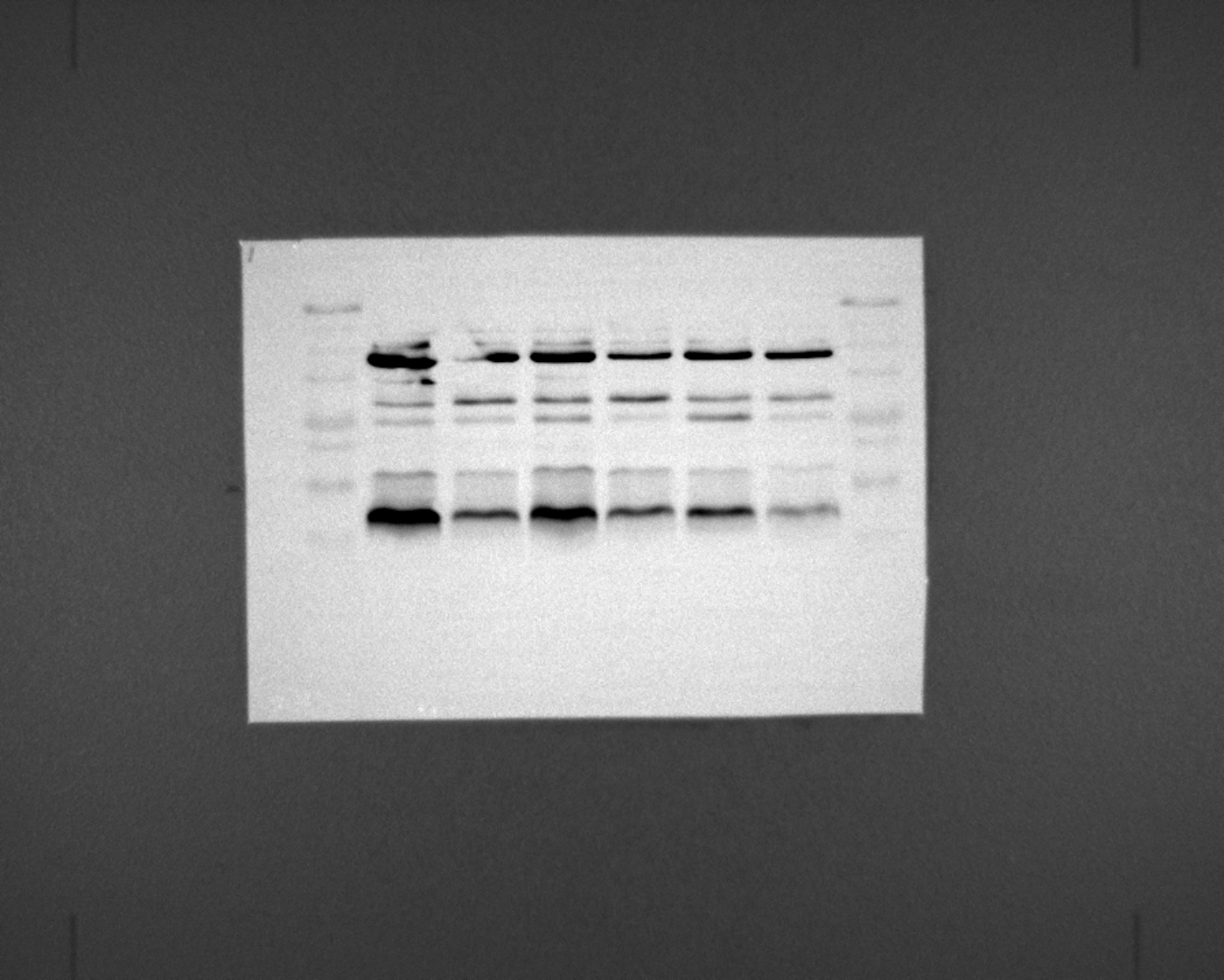

Supplement: Supplementary file 1 [file DataSheet1.ZIP › supplementary/figure5/5F/cytoC M.tif]

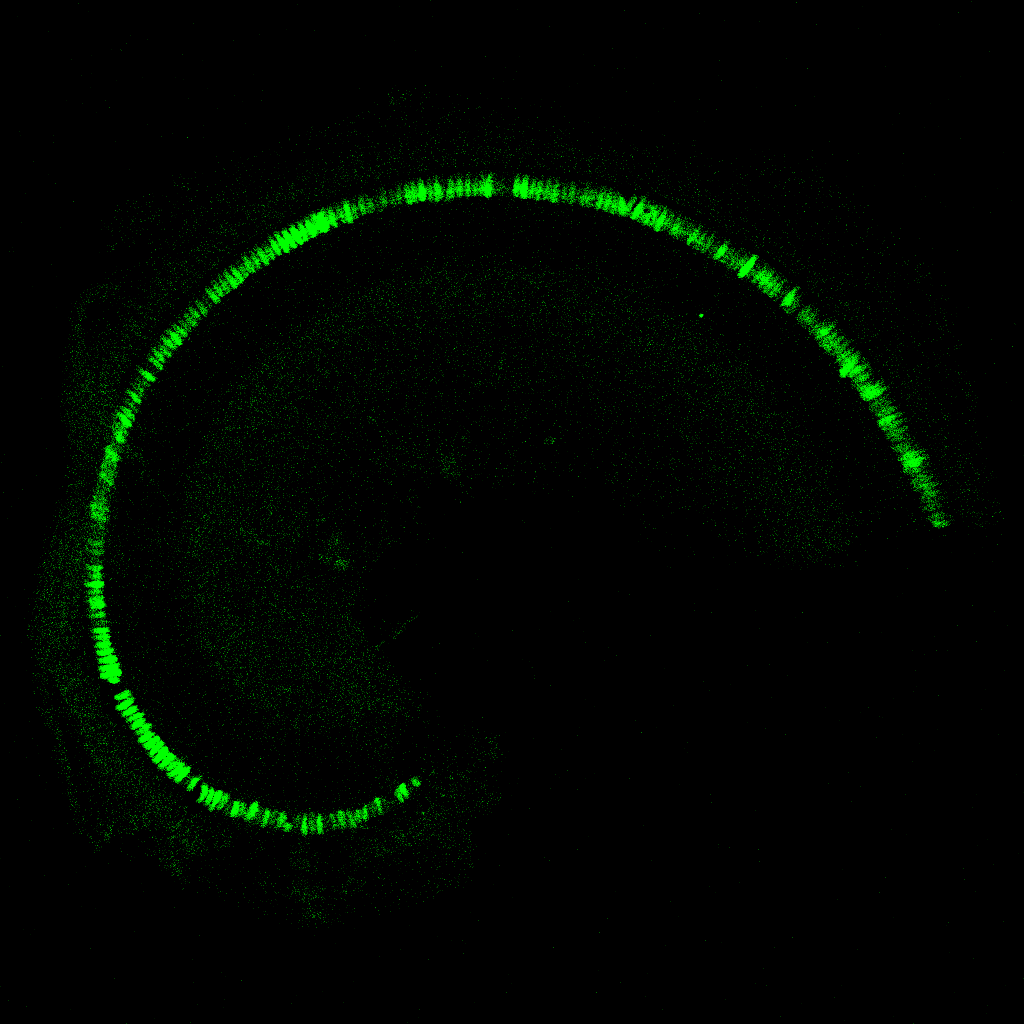

Supplement: Supplementary file 1 [file DataSheet1.ZIP › supplementary/figure6/6B.tif]

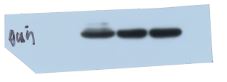

Supplement: Supplementary file 1 [file DataSheet1.ZIP › supplementary/figure6/6C/actin.JPG]

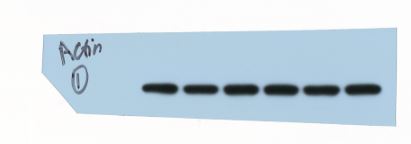

Supplement: Supplementary file 1 [file DataSheet1.ZIP › supplementary/figure6/6C/BA.JPG]

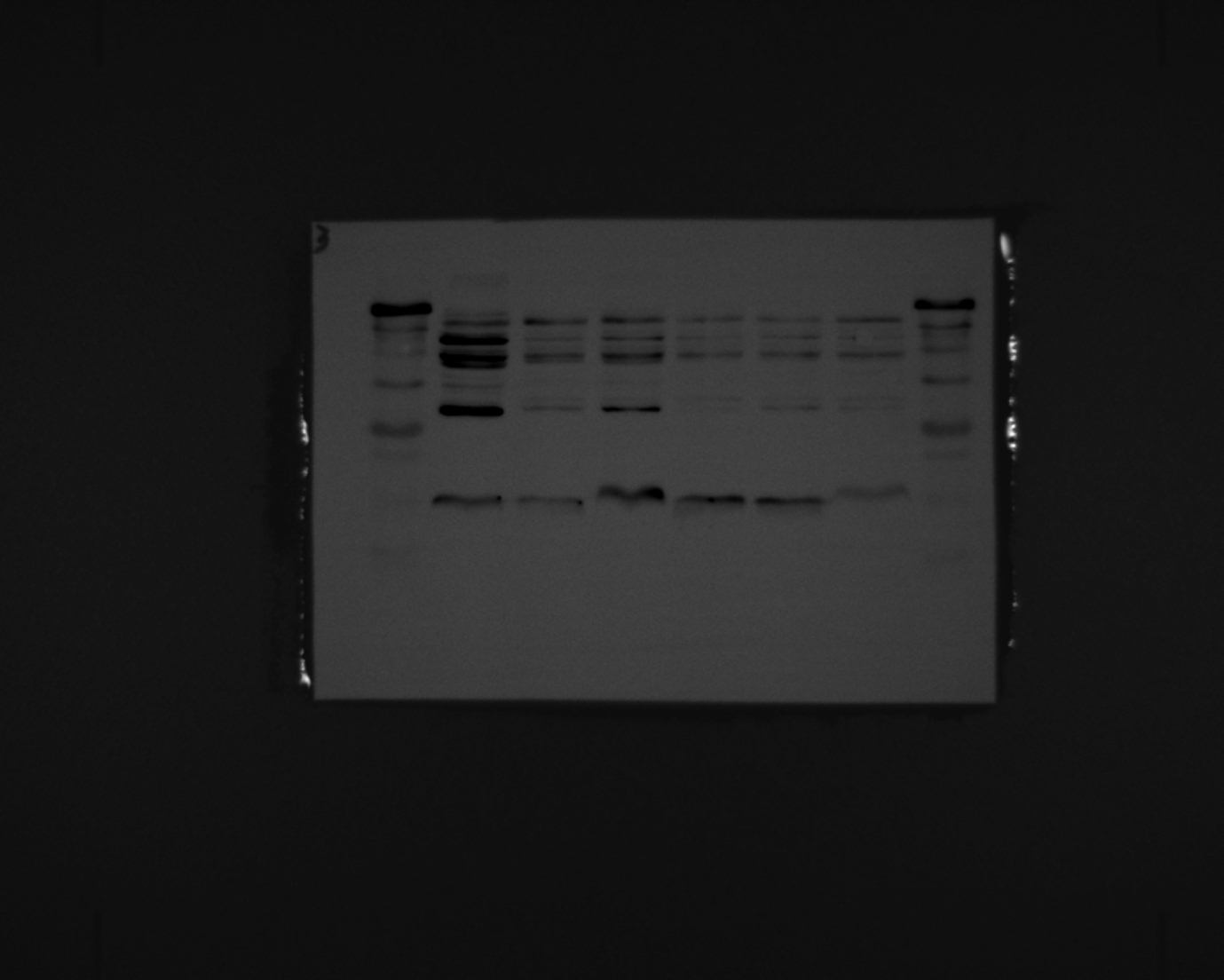

Supplement: Supplementary file 1 [file DataSheet1.ZIP › supplementary/figure6/6C/SIRT3 M.jpg]

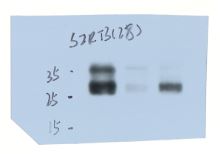

Supplement: Supplementary file 1 [file DataSheet1.ZIP › supplementary/figure6/6C/SIRT3-1.JPG]

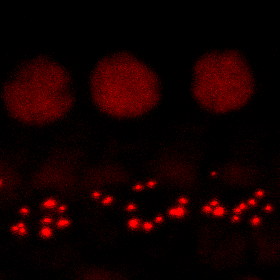

Supplement: Supplementary file 1 [file DataSheet1.ZIP › supplementary/figure6/6E/apical/PTS+2ME.tif]

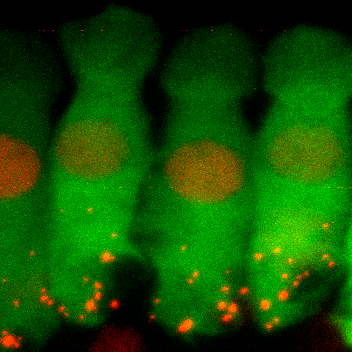

Supplement: Supplementary file 1 [file DataSheet1.ZIP › supplementary/figure6/6E/apical/PTS+AAV+2ME.tif]

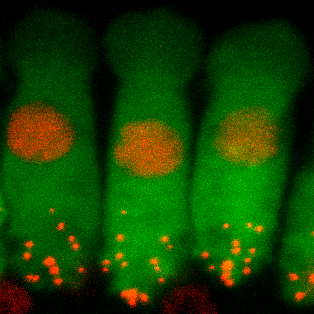

Supplement: Supplementary file 1 [file DataSheet1.ZIP › supplementary/figure6/6E/apical/PTS+AAVSIRT3.tif]

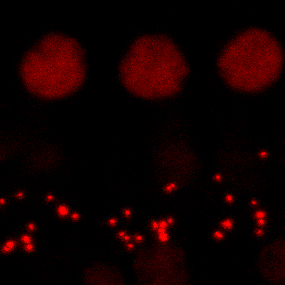

Supplement: Supplementary file 1 [file DataSheet1.ZIP › supplementary/figure6/6E/apical/PTS.tif]

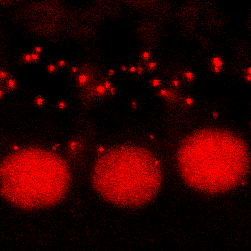

Supplement: Supplementary file 1 [file DataSheet1.ZIP › supplementary/figure6/6E/basal/PTS+2ME.tif]

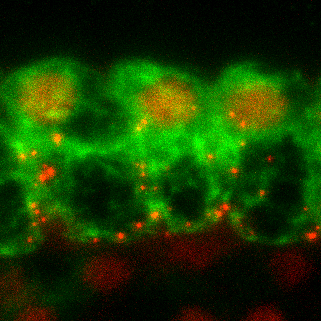

Supplement: Supplementary file 1 [file DataSheet1.ZIP › supplementary/figure6/6E/basal/PTS+AAV+2ME.tif]

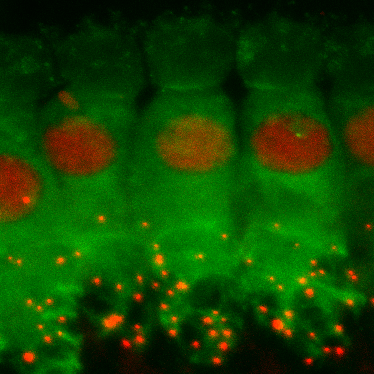

Supplement: Supplementary file 1 [file DataSheet1.ZIP › supplementary/figure6/6E/basal/PTS+AAV.tif]

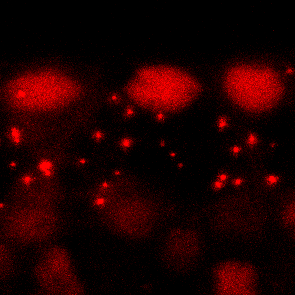

Supplement: Supplementary file 1 [file DataSheet1.ZIP › supplementary/figure6/6E/basal/PTS.tif]

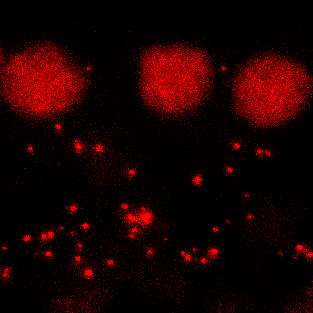

Supplement: Supplementary file 1 [file DataSheet1.ZIP › supplementary/figure6/6E/MIDDLE/PTS+2ME.tif]

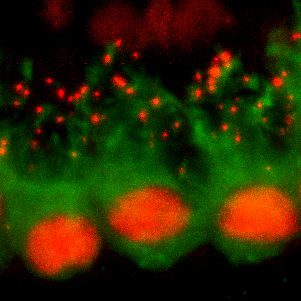

Supplement: Supplementary file 1 [file DataSheet1.ZIP › supplementary/figure6/6E/MIDDLE/PTS+AAV+2ME.tif]

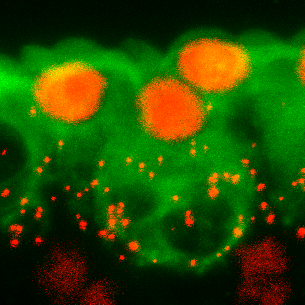

Supplement: Supplementary file 1 [file DataSheet1.ZIP › supplementary/figure6/6E/MIDDLE/PTS+AAV.tif]

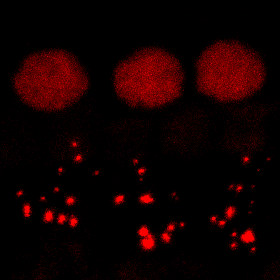

Supplement: Supplementary file 1 [file DataSheet1.ZIP › supplementary/figure6/6E/MIDDLE/PTS.tif]
